# Hepatitis C Virus Proteins Interact with the Endosomal Sorting Complex Required for Transport (ESCRT) Machinery via Ubiquitination To Facilitate Viral Envelopment

**DOI:** 10.1128/mBio.01456-16

**Published:** 2016-11-01

**Authors:** Rina Barouch-Bentov, Gregory Neveu, Fei Xiao, Melanie Beer, Elena Bekerman, Stanford Schor, Joseph Campbell, Jim Boonyaratanakornkit, Brett Lindenbach, Albert Lu, Yves Jacob, Shirit Einav

**Affiliations:** a Division of Infectious Diseases and Geographic Medicine, Department of Medicine, and Department of Microbiology and Immunology, Stanford University School of Medicine, Stanford, California, USA; b Department of Microbial Pathogenesis, Yale School of Medicine, New Haven, Connecticut, USA; c Department of Biochemistry, Stanford University School of Medicine, Stanford, California, USA; d Département de Virologie, Unité de Génétique Moléculaire des Virus ARN (GMVR), Institut Pasteur, Centre national de la recherche scientifique, and Université Paris Diderot, Paris, France

## Abstract

Enveloped viruses commonly utilize late-domain motifs, sometimes cooperatively with ubiquitin, to hijack the endosomal sorting complex required for transport (ESCRT) machinery for budding at the plasma membrane. However, the mechanisms underlying budding of viruses lacking defined late-domain motifs and budding into intracellular compartments are poorly characterized. Here, we map a network of hepatitis C virus (HCV) protein interactions with the ESCRT machinery using a mammalian-cell-based protein interaction screen and reveal nine novel interactions. We identify HRS (hepatocyte growth factor-regulated tyrosine kinase substrate), an ESCRT-0 complex component, as an important entry point for HCV into the ESCRT pathway and validate its interactions with the HCV nonstructural (NS) proteins NS2 and NS5A in HCV-infected cells. Infectivity assays indicate that HRS is an important factor for efficient HCV assembly. Specifically, by integrating capsid oligomerization assays, biophysical analysis of intracellular viral particles by continuous gradient centrifugations, proteolytic digestion protection, and RNase digestion protection assays, we show that HCV co-opts HRS to mediate a late assembly step, namely, envelopment. In the absence of defined late-domain motifs, K63-linked polyubiquitinated lysine residues in the HCV NS2 protein bind the HRS ubiquitin-interacting motif to facilitate assembly. Finally, ESCRT-III and VPS/VTA1 components are also recruited by HCV proteins to mediate assembly. These data uncover involvement of ESCRT proteins in intracellular budding of a virus lacking defined late-domain motifs and a novel mechanism by which HCV gains entry into the ESCRT network, with potential implications for other viruses.

## IMPORTANCE

Viruses commonly bud at the plasma membrane by recruiting the host ESCRT machinery via conserved motifs termed late domains. The mechanism by which some viruses, such as HCV, bud intracellularly is, however, poorly characterized. Moreover, whether envelopment of HCV and other viruses lacking defined late domains is ESCRT mediated and, if so, what the entry points into the ESCRT pathway are remain unknown. Here, we report the interaction network of HCV with the ESCRT machinery and a critical role for HRS, an ESCRT-0 complex component, in HCV envelopment. Viral protein ubiquitination was discovered to be a signal for HRS binding and HCV assembly, thereby functionally compensating for the absence of late domains. These findings characterize how a virus lacking defined late domains co-opts ESCRT to bud intracellularly. Since the ESCRT machinery is essential for the life cycle of multiple viruses, better understanding of this virus-host interplay may yield targets for broad-spectrum antiviral therapies.

## INTRODUCTION

To acquire their envelope, viruses bud at the plasma membrane and/or intracellularly in a process that involves membrane curving and fission. This budding topology (away from cytoplasm, unlike endocytic vesicles) is equivalent to that of vesicle budding into multivesicular bodies (MVBs), which is mediated by the endosomal sorting complex required for transport (ESCRT) machinery. Indeed, the ESCRT machinery is critical for envelopment of multiple RNA viruses that bud at the plasma membrane, including retroviruses, filoviruses, arenaviruses, and rhabdoviruses (1, 2). Nevertheless, the role of ESCRT in the less common, intracellular form of budding, characteristic of some RNA viruses such as *Flaviviridae*, is poorly characterized.

The ESCRT machinery is composed of five protein complexes (ESCRT-0, ESCRT-I, ESCRT-II, ESCRT-III, and VPS/VTA1) and associated proteins. These complexes act sequentially to recruit and cluster cargo proteins (ESCRT-0), curve membranes (ESCRT-I and ESCRT-II), catalyze vesicle fission (ESCRT-III and VPS/VTA1), and disassemble the ESCRT-III complex (VPS/VTA1) (1).

K63-linked polyubiquitination is the main recognition signal of host cargo proteins used by ESCRT components for sorting into the endosomal pathway (1, 3, 4). This interaction is mediated by ubiquitin-binding domains, such as the ubiquitin-interacting motif (UIM), within the ESCRT-0 complex subunits, HRS (hepatocyte growth factor-regulated tyrosine kinase substrate) and STAM1/2. Viruses recruit the ESCRT machinery via late domains, conserved motifs within viral structural proteins. Among the defined late domains are the P(T/S)AP, YPXL, and PPXY signals, which bind TSG101 (ESCRT-I), Alix (an accessory protein), and NEDD4 family proteins (E3 ligases), respectively (2). Ubiquitin cooperates with these late domains to facilitate ESCRT-mediated budding of some viruses (4). However, whether envelopment of viruses lacking defined late domains is ESCRT mediated and how such viruses gain entry into the ESCRT pathway remain unknown.

Beyond viral budding, the ESCRT machinery has been implicated in the release of the nonenveloped RNA viruses hepatitis A virus (HAV) and bluetongue virus (BTV) (5, 6) and the assembly of replication complexes of tomato bushy stunt virus (TBSV) and brome mosaic virus (BMV) in the peroxisome lumen and endoplasmic reticulum (ER), respectively (7, 8). ESCRT components are also hijacked by DNA viruses, such as herpesviruses, to mediate nuclear egress and secondary envelopment (9) and by HBV to facilitate intracellular budding and/or release (10).

The *Flaviviridae* is a family of enveloped, positive, single-stranded RNA viruses that includes hepatitis C virus (HCV), a major cause of liver disease. The HCV core protein and E1 and E2 (E1-E2) glycoproteins form new virions; the nonstructural (NS) proteins NS3, NS4A, NS4B, NS5A, and NS5B form the viral replication machinery, whereas p7 and NS2 are essential for infectious virus production (11–13). The current model of HCV assembly suggests that viral particles begin to assemble on or near the surface of lipid droplets (LDs), where core is concentrated (14). Similarly to flaviviruses, HCV is thought to bud into the ER, where the envelope proteins are retained. HCV particles, rendered infectious upon budding, exit the cell via the secretory pathway. HCV assembly requires coordination of all 10 HCV proteins along with multiple host factors (14). Moreover, NS2, in particular, is critical in bringing together the viral components required for HCV envelopment, while p7 coordinates this step (11, 12, 15–18). Nevertheless, a comprehensive understanding of the virus-host interplay underlying HCV envelopment is still lacking.

Prior work suggested that ESCRT proteins are essential for infectious HCV production, particularly viral release (19–21). However, their precise role remains to be elucidated. Moreover, since inspection of the HCV polyprotein sequence reveals no defined late domains, the mechanism by which HCV recruits the ESCRT machinery remains unknown. We hypothesized that HCV proteins recruit ESCRT components via ubiquitination to mediate HCV envelopment. To test this hypothesis, we screened for HCV interactions with 24 ESCRT proteins by mammalian-cell-based protein-fragment complementation assays (PCAs) (22). Nine novel interactions were identified, and HRS emerged as a critical entry point into the ESCRT network. We demonstrated that K63 polyubiquitination of NS2 lysine residues mediates binding to HRS UIM and HCV assembly, thereby compensating for the apparent absence of late domains. Furthermore, we show that HRS is an important factor for HCV envelopment and that ESCRT-III and VPS/VTA1 factors are also hijacked by HCV core and NS5A, respectively, to mediate viral assembly.

## RESULTS

### Interactions between HCV proteins and ESCRT components measured by PCAs.

**FIG 1:**
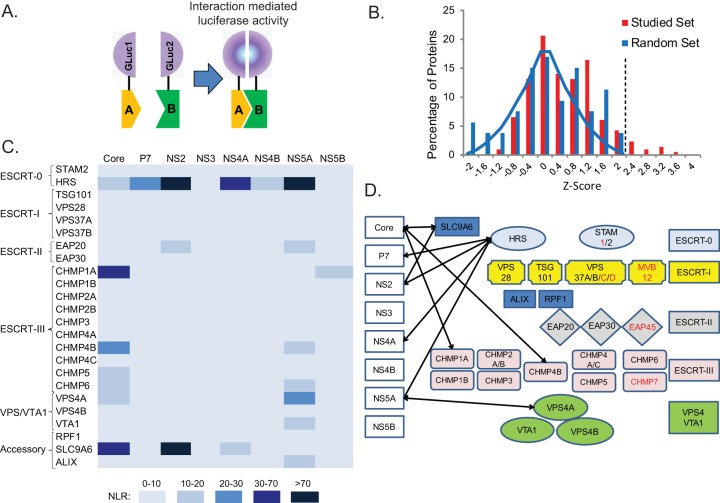
Interactions between HCV proteins and ESCRT components measured by PCAs. (A) Schematics of the PCA format. Letters A and B in panel A represent prey and bait proteins fused to GLuc1 and GLuc2 luciferase fragments. (B) Histogram of the mean *z* score values of the studied set and random reference set (RRS) of interactions obtained from 3 independent experiments. The blue line represents a Gaussian fit to the RRS *z* scores. The dotted line defines the cutoff used for positive interactions. (C) A heat map of the interactions color coded on the basis of the NLR. (D) Model for the HCV-ESCRT interaction network detected in the screen. ESCRT components labeled red were unavailable in the ORFeome library.

In search of factors that provide access to the ESCRT machinery, we screened for interactions between a panel of ESCRT proteins and the HCV proteome using PCAs (excluding E1 and E2, which form disulfide-linked misfolded aggregates when ectopically coexpressed in cells [23]). This PCA format relies on reversible reconstitution of a split luciferase reporter (Fig. 1A) and provides a high-fidelity means to measure weak and transient interactions such as those between ESCRT and cargo (with dissociation constants [*K_d_*s] in the micromolar range) (22, 24, 25). Moreover, it allows detection of interactions involving membrane proteins with posttranslational modifications, such as ubiquitination, in mammalian cells and within appropriate subcellular compartments. A total of 24 ESCRT protein-coding genes were selected from the human ORFeome library (26) and fused to an N-terminal luciferase fragment reporter (GLuc1-A; letter A in Fig. 1A), while individual HCV proteins derived from the J6/JFH genome (27) were fused to an N-terminal complementary luciferase fragment (GLuc2-B; letter B in Fig. 1A). ESCRT and viral genes were transfected pairwise into 293T cells. When screening for NS3-ESCRT interactions, a plasmid encoding FLAG-tagged NS4A was added to allow membrane binding of the NS3 protein (28). Expression of the viral (see Fig. S1 in the supplemental material) and ESCRT (data not shown) proteins was confirmed by Western blotting. Luciferase activity was measured at 24 h posttransfection, and results are expressed as normalized luminescence ratios (NLR). We benchmarked the accuracy and sensitivity of this screen by analysis of a random reference set (RRS) composed of 53 noninteracting human protein pairs (22). *z* scores indicating the numbers of standard deviations (SDs) above the mean NLR of the control RRS were calculated (see Tables S1 and S2). A histogram distribution curve of the mean *z* score values obtained from three independent experiments demonstrated a clear separation between the studied set and the RRS (*P* = 5.9 × 10^−6^, *t* test) (Fig. 1B). An SD cutoff value of >2.2 (corresponding to an NLR of >25) was chosen as the threshold to define positive interactions. A positive-control interaction between HRS and TSG101 and the majority of a set of 17 already-known HCV-host protein interactions yielded *z* scores at or above the cutoff, as expected (see Table S3). Nine novel interactions were identified and categorized based on their NLR (Fig. 1C). Six of these interactions involved either HRS, which bound four of the eight studied HCV proteins with the highest apparent affinity, or its regulator, SLC9A6 (29) (Fig. 1D). In addition, ESCRT-III proteins CHMP1A and CHMP4B both interacted with core and VPS4A interacted with NS5A.

### Validation of HRS interactions with HCV proteins.

**FIG 2:**
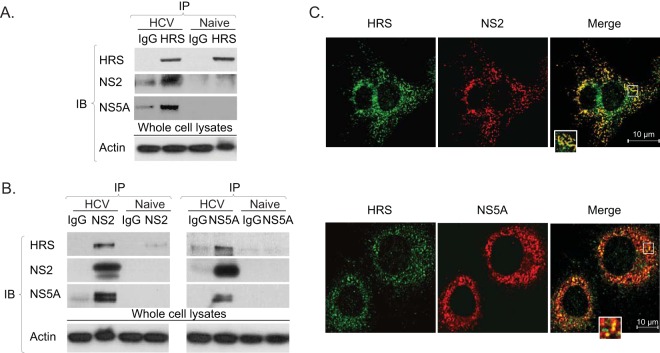
HRS interacts with NS2 and NS5A in HCV-transfected cells. (A and B) Immunoprecipitations (IPs) from membrane fractions of HCV-transfected Huh-7.5 cells and naive cells with anti-HRS antibodies (A) or anti-NS2 or anti-NS5A antibodies (B). Immunoblotting (IB) antibodies are listed on the left. (C) Representative confocal IF microscopy images at ×60 magnification of HRS (green) and NS2 and NS5A (red) in HCV-transfected cells. *n* = >20.

HRS emerged as a key binding partner of several HCV proteins in the PCA screen, consistent with its ability to form multivalent interactions with numerous cargo proteins (24). We subsequently validated the highest-apparent affinity interactions of HRS with NS2 and NS5A in the context of HCV infection. Coimmunoprecipitations (co-IP) were conducted in membrane fractions derived from cells transfected with J6/JFH HCV RNA and untransfected (naive) controls. Anti-HRS antibody effectively pulled down NS2 and NS5A, whereas only background signal was demonstrated with IgG controls (Fig. 2A). Reciprocal co-IPs revealed that anti-NS2 and anti-NS5A antibodies, but not an IgG control, pulled down HRS (Fig. 2B). Moreover, anti-NS2 antibodies pulled down NS5A and anti-NS5A antibodies pulled down NS2, suggesting that HRS is associated with NS2 and NS5A in cocomplexes. Lack of signal in IPs from naive cells (Fig. 2A and B) confirmed the specificity of viral protein detection in the HCV RNA-transfected cells. In addition, significant colocalization of NS2 and NS5A with HRS was observed by confocal immunofluorescence (IF) analysis of 20 to 40 HCV-transfected cells (Manders’ colocalization coefficients of 84% ± 8% and 58% ± 10%, respectively) (Fig. 2C).

### HRS is essential for HCV assembly.

**FIG 3:**
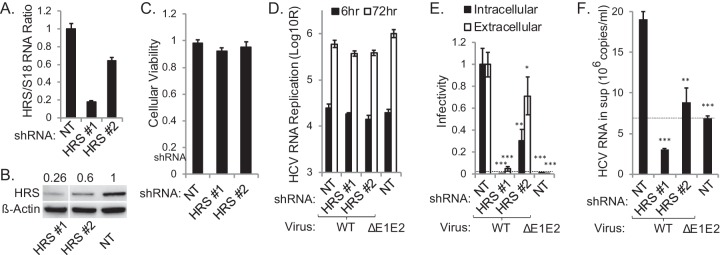
HRS is essential for HCV assembly. (A) qRT-PCR quantification of HRS transcript normalized to 18S. (B) HRS protein normalized to actin (numbers represent HRS-to-actin protein ratios relative to the NT control). Non-consecutive lanes ran on the same gel are shown. (C) Relative cell viability in cell lines stably expressing indicated shRNAs. (D) HCV RNA replication in stable cell lines 6 and 72 h postelectroporation with WT HCV RNA or E1-E2 deletion mutant measured by luciferase assays (RLU = relative light units). (E) Intra- and extracellular infectivity measured by luciferase assays in naive cells inoculated with clarified cell lysates and supernatants derived from the HCV-electroporated cell lines, respectively. Plotted data represent infectivity normalized to NT controls. (F) Level of viral RNA released into the supernatant (sup) at 72 h postelectroporation. Dashed lines represent the background level observed in the ΔE1-E2 mutant. Means ± SD of results of 3 independent experiments are shown. *, *P* < 0.05; **, *P* < 0.01; ***, *P* < 0.001 (Student’s *t* test).

To determine the functional relevance of HRS to HCV infection, we established Huh-7.5 cell lines stably expressing short hairpin RNA (shRNA) targeting HRS or a nontargeting (NT) sequence and transfected them with J6/JFH(p7-Rluc2A) RNA, a luciferase reporter virus (30). Effective suppression of HRS was achieved (Fig. 3A and B), without apparent cytotoxic effects (Fig. 3C). HRS depletion had no effect on HCV RNA replication, as measured by luciferase assays at 6 and 72 h posttransfection (Fig. 3D). Nevertheless, inoculation of naive cells with clarified cell lysates and supernatants derived from the HCV-transfected cells resulted in up to ~20-fold reductions in both intra- and extracellular infectivity, respectively, in HRS-depleted cells relative to NT controls (Fig. 3E). An assembly-defective (ΔE1-E2) virus infection produced luciferase signals below the background level. These results indicate a severe assembly defect which correlates with the level of HRS suppression and are in agreement with a former study (21). The level of HCV RNA released into supernatants of HRS-depleted cells was comparable to or lower than that released by the ΔE1-E2 mutant, excluding the possibility of production of defective, noninfectious RNA-containing particles (30) (Fig. 3F). A similar effect on HCV assembly was demonstrated in cells transiently depleted of HRS by pooled small interfering RNAs (siRNAs) (see Fig. S2A to F in the supplemental material).

To determine whether the role of HRS in HCV assembly correlates with alterations in its subcellular localization, we performed a quantitative confocal IF analysis. While only 20% ± 15% of Bodipy-labeled LD stained positive for HRS in naive Huh-7.5 cells, 72% ± 17% of LD were HRS positive 72 h following HCV transfection (*P* value < 0.001) (see Fig. S2G in the supplemental material). Similarly, 30% ± 8% of calnexin-labeled ERs were HRS positive in naive cells, whereas 56% ± 7% were positive in HCV-transfected cells (*P* value < 0.001) (see Fig. S2H). Together, these results suggest that HCV recruits HRS to LDs and the ER to mediate viral assembly.

### HRS mediates HCV envelopment.

**FIG 4:**
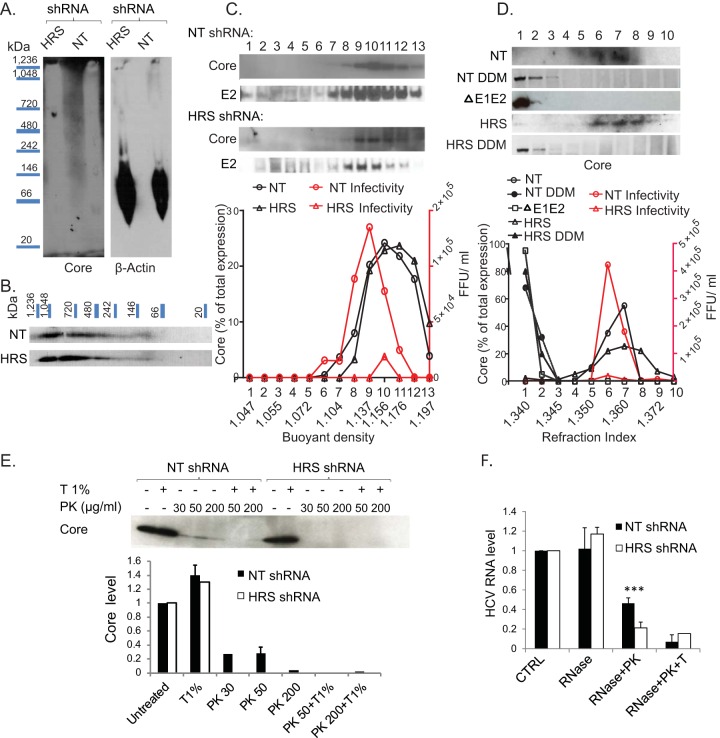
HRS mediates HCV envelopment. Lysates of HCV-transfected HRS-depleted cell lines or NT controls were subjected to two-dimensional gel electrophoresis. Membranes were blotted with anti-core and anti-actin antibodies. (A and B) Separation of core complexes by BN-PAGE (A) and denaturing SDS-PAGE (B). The experiment was conducted twice. (C and D) Sucrose density gradient analysis of intracellular viral particles derived from HCV-transfected cell lines depleted of HRS by shRNA or NT controls by isopycnic (C) and rate-zonal-like (D) separations in the presence or absence of 1% DDM. Thirteen and ten fractions were collected, respectively, and analyzed. Each experiment was conducted three times. Representative membranes blotted with anti-core and anti-E2 antibodies are shown. Plotted data represent the core content along the gradient normalized to the total core protein and levels of infectivity (in numbers of focus-forming units [FFU] per milliliter). ΔE1-E2, E1-E2 deletion HCV mutant. (E) Proteolytic digestion protection assays of lysates of HCV-transfected cell lines depleted of HRS or NT controls. A representative membrane is shown, demonstrating the amount of protease-resistant core following no treatment or treatment with Triton (T) and/or PK. Plotted data represent relative core levels from 2 experiments quantified by Western blotting and normalized to untreated controls. (F) Fraction 10 of the isopycnic gradient (see panel A) was subjected to RNase digestion protection assays. Samples were left untreated or were treated with S7 RNase alone, with S7 RNase following PK treatment, or with S7 RNase, Triton (T), and PK. Plotted data represent relative residual HCV RNA levels normalized to untreated controls.

To pinpoint the precise role of HRS in HCV assembly, we first determined the effect of HRS depletion on core oligomerization. Lysates of HCV RNA-transfected cells were separated by blue native polyacrylamide gel electrophoresis (BN-PAGE). Core signal could not be detected in the blot possibly due to failure of large assembled capsids to enter the gel and/or masking of core epitopes within these complexes (Fig. 4A). To dissociate large core complexes and enable detection, we conducted two-dimensional electrophoresis, as previously described (15, 31). The individual lanes cut out of the BN-PAGE gel (first dimension) were stacked perpendicularly in a large single well followed by denaturing SDS-PAGE separation (second dimension). This analysis revealed a variety of core complexes (~100 to >1,240 kDa) (Fig. 4B), representing intermediate core complexes and likely fully assembled capsids, as previously reported (15). Notably, a similar pattern of distribution of core complexes was detected in HRS-depleted samples, suggesting that HRS does not affect core oligomerization.

Next, to determine whether the capsid is enveloped upon HRS depletion, we characterized the density of intracellular viral particles by isopycnic separation. Clarified lysates of HCV-transfected HRS-depleted or control NT cell lines were layered on top of a sucrose gradient (10% to 60%) and spun for 16 h. Thirteen fractions were collected and subjected to measurement of buoyant density levels by a refractometer, core and E2 levels by immunoblotting, infectivity levels by focus formation assays, and HCV RNA levels by quantitative reverse transcription-PCR (qRT-PCR). The peak of core determined by immunoblotting coincided with the peak of infectivity in NT samples (Fig. 4C). Although HRS depletion suppressed infectivity, it did not alter core’s distribution along the gradient (Fig. 4C) or its cosedimentation with either the E2 glycoprotein (Fig. 4C) or HCV RNA (see Fig. S3 in the supplemental material). The density of intracellular particles in fractions harboring the peak of core in NT and HRS-depleted samples was 1.15 to 1.17 g/ml (Fig. 4C), consistent with a previous report (32).

To study the size and mass of intracellular viral particles, clarified lysates of HCV-transfected HRS-depleted or NT control cell lines were layered on top of a sucrose density gradient (0% to 30%) and spun for 1 h. These hybrid separations performed much like rate-zonal gradients, although the gradients included heavier fractions (i.e., fractions 9 and 10) incapable of separating low-density particles based solely on their velocity of sedimentation. A total of 10 fractions were collected and analyzed as described above. There was a prominent peak of core in fractions 6 and 7 of NT controls transfected with wild-type (WT) HCV, in agreement with Gentzch et al. (15) (Fig. 4D). Notably, core shifted to fractions 1 and 2 upon disruption of the lipid envelope with detergent (1% DDM [*n*-dodecyl-β-d-maltopyranoside]) or transfection with nonenveloping ΔE1-E2 HCV mutant (Fig. 4D). In analogy to the NT samples, core sedimented in fractions 6 to 8 in HRS-depleted samples and shifted to fractions 1 and 2 upon detergent treatment (Fig. 4D). The peak of infectivity coincided with the peak of core in both NT and HRS-depleted samples (Fig. 4D). These data suggest that fractions 6 and 7 carry the bulk of infectious particles, whereas the core protein species sedimenting in fractions 1 and 2 may represent nonenveloped particles. Nevertheless, the overall infectivity of HRS-depleted samples was significantly reduced despite the prominent sedimentation of core protein in the relevant fractions (Fig. 4D).

Taking the data together, while HRS depletion causes a severe defect in intracellular infectivity, it does not increase accumulation of nonenveloped capsids and/or oligomeric core. Thus, HRS may be essential during a late assembly step by mediating completion of envelopment or a postbudding step critical for infectivity.

To distinguish between these possibilities, we monitored envelope protection of the capsid by examining the resistance of core to proteinase K (PK) digestion. Lysates derived from HRS-depleted or NT control cells transfected with J6/JFH HCV were left untreated or were treated with 1% Triton and/or PK. Residual core protein was quantified by Western blotting. While treatment with Triton alone did not affect core abundance, treatment with PK alone resulted in core proteolysis in the NT control samples (Fig. 4E). Nevertheless, a fraction of core remained protected from the protease in the NT samples, consistent with an intact envelope. As predicted, core underwent near complete proteolysis by PK following pretreatment with Triton in NT samples. In lysates derived from HRS-depleted cells, core was unaffected by Triton treatment alone; however, even a low concentration of PK alone was sufficient to completely degrade core (Fig. 4E).

To further characterize the aberrant intracellular HCV particles, we examined their ability to protect the viral genome from RNase digestion. Fraction 10 of the isopycnic gradient was left untreated or subjected to treatment with S7 RNase alone, with S7 RNase following pretreatment with PK, or with S7 RNase following pretreatment with Triton plus PK. Residual HCV RNA was quantified by quantitative PCR (qPCR). Viral RNA in the NT samples was fully protected from RNase following RNase treatment, partially protected from RNase following pretreatment with PK, and fully susceptible to RNase following pretreatment with Triton and PK (Fig. 4F). HCV RNA was similarly protected in HRS-depleted samples when RNase was used alone, further supporting assembly of intact nucleocapsids. However, there was a nearly complete loss of the protected viral RNA fraction upon pretreatment with PK that was comparable to the background measured in samples pretreated with Triton and PK. Together, these results indicate that although a viral envelope appears to be associated with assembling virions within HRS-depleted cells, it is functionally impaired at protecting the capsid and viral genome, thereby supporting the idea of a role for HRS in completion of envelopment.

### HRS binds HCV proteins via its UIM.

**FIG 5:**
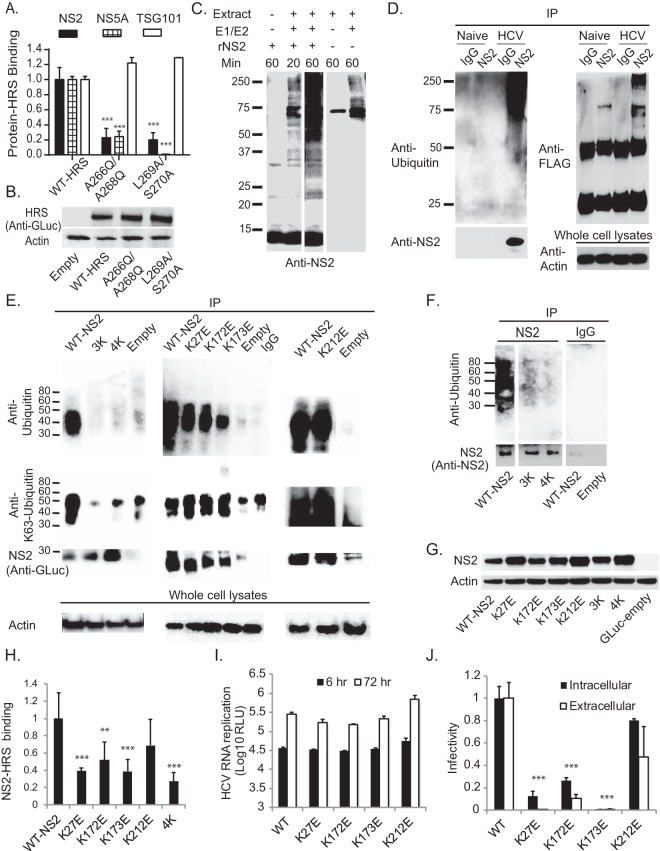
NS2 K63-ubiquitinated lysine residues mediate HRS UIM binding and HCV assembly. (A) Analysis of interactions of WT and mutant HRS with NS2, NS5A, and TSG101 by PCAs. Data are presented as NLRs relative to the interaction with WT HRS. (B) Levels of HRS in lysates of cells transfected with the indicated plasmids. (C) Purified recombinant truncated NS2 (rNS2) (92 to 216 aa) was incubated with ubiquitin-hemagglutinin (UB-HA) for 20 or 60 min in the presence or absence of E1 and E2 enzymes and Huh-7.5 cell extract. Cell extracts incubated in the absence of NS2 served as controls. A representative membrane blotted with anti-NS2 antibodies is shown. Samples were run on the same gel, from which two lanes were cut out. (D) Lysates of HCV-transfected or naive Huh-7.5 cells were incubated with FLAG anti-K63 TUBE reagent followed by IP with anti-NS2 antibodies or IgG. Representative membranes blotted with antibodies against NS2, ubiquitin, FLAG, and actin are shown. (E and F) Huh-7.5 cells were cotransfected with WT or mutant GLuc-NS2 and ubiquitin and subjected to IP with anti-GLuc (E) or anti-NS2 (F) antibodies or IgG under denaturing conditions. Membranes were blotted with antibodies against ubiquitin, polyubiquitin-K63, GLuc (E), NS2 (F), and actin. Nonconsecutive lanes that were run on the same gel are shown in panel F. (G) Levels of NS2 in lysates of cells transfected with the indicated plasmids. (H) Analysis of interactions of WT or mutant NS2 with HRS by PCAs. Plotted data represent NLRs relative to WT NS2-HRS binding. (I) Cells were electroporated with WT or mutated NS2 bicistronic J6/H77NS2/JFH HCV RNA. HCV RNA replication was measured by luciferase assays at 6 and 72 h postelectroporation, and results are expressed as RLU. (J) Intra- and extracellular infectivity analyzed by luciferase assays in naive cells inoculated with clarified cell lysates and supernatants derived from the HCV-electroporated cells, respectively. Plotted data represent infectivity normalized to WT controls. Mean values and standard deviations of results of 3 independent experiments are shown. *, *P* < 0.05; **, *P* < 0.01; ***, *P* < 0.001 (Student’s *t* test).

Next, we set out to decipher the mechanism by which HRS interacts with HCV proteins. To test our hypothesis that HRS recognizes ubiquitinated residues within HCV proteins via its UIM, we introduced UIM double mutations that impair ubiquitin binding, A266Q/A268Q and L269A/S270A (24). These mutations disrupted HRS binding to NS2 and NS5A as measured by PCAs (Fig. 5A) without altering HRS expression (Fig. 5B) or (PSAP-mediated) TSG101 binding (1) (Fig. 5A). These results suggest that HRS UIM is involved in mediating its binding to HCV proteins.

### NS2 undergoes ubiquitination *in vitro* and in HCV-transfected cells.

We then sought to identify ubiquitination sites in HCV proteins that may mediate their binding to HRS UIM. HCV core (33), NS5A (34), and E2 (35) are ubiquitinated. However, since their ubiquitination is also implicated in protein degradation, mutating the relevant lysine residues increases protein levels, thereby hindering assessment of other potential functions of these residues. NS2 has not been shown to be ubiquitinated to date. Interestingly, mutations in NS2 lysine residues impair HCV assembly with no effect on NS2 expression (16, 36, 37). We hypothesized that NS2 is ubiquitinated and that NS2-ubiquitinated lysines mediate HRS binding and HCV assembly.

To test this hypothesis, we first conducted *in vitro* ubiquitination assays. Truncated recombinant NS2 protein (rNS2) (92 to 216 amino acids [aa]), previously shown to be proteolytically active (38, 39), fused to a C-terminal glutathione *S*-transferase (GST) tag was expressed in *Escherichia coli* and purified on glutathione beads (40) (see Fig. S4A to C in the supplemental material). Following cleavage of the GST tag, rNS2 was incubated for 20 to 60 min with ubiquitin in the presence or absence of E1 and E2 (UBCH2) enzymes and Huh-7.5 cell extract (source of E3 ligases). Samples were separated by SDS-PAGE and membranes blotted with anti-NS2 antibodies. In the absence of E1, E2, and E3 enzymes, a prominent ~14-kDa band was observed, corresponding to recombinant NS2. Addition of E1 and E2 enzymes and cell extract resulted in accumulation of multiple bands (25 to >250 kDa) with increased intensity over time (Fig. 5C). In the absence of NS2, cell extract controls displayed a nonspecific ~75-kDa band when incubated for 1 h with ubiquitin and a background level of laddering (75 to >250 kDa) upon addition of the E1 and E2 enzymes. The very high signal-to-noise ratio in the 75- to >250-kDa range and additional laddering displayed at <70 kDa following a 1-h incubation with NS2 indicate specific signal in the presence of NS2. These results suggest that NS2 is modified by ubiquitination.

Next, we studied NS2 ubiquitination in the context of HCV infection. Lysates derived from Huh-7.5 cells transfected with Jc1 HCV RNA (41) or naive cells were subjected to IP under denaturing conditions (see Fig. S4D in the supplemental material) (42). NS2 antibody but not IgG controls effectively pulled down NS2 (see Fig. S4D). In addition to an ~25-kDa band corresponding to monomeric NS2, samples derived from HCV RNA-transfected cells but not naive cells displayed a smear of bands (~80 to 250 kDa) that stained with both anti-NS2 and anti-ubiquitin antibodies (see Fig. S4D). These results suggest that NS2 is polyubiquitinated in the context of HCV infection. Since HRS binds K63-linked polyubiquitin (3), we then tested whether NS2 undergoes specifically K63-linked polyubiquitination. To do so, samples derived from HCV RNA-transfected cells and naive cells were incubated for 1 h with FLAG anti-K63 TUBE (tandem ubiquitin binding entity) (43), a reagent consisting of FLAG-tagged UIMs joined by a linker, previously shown to selectively bind K63-linked polyubiquitin. Samples were then subjected to IP with anti-NS2 or IgG antibodies, and membranes were blotted with anti-NS2, ubiquitin, and FLAG antibodies (Fig. 5D). Effective pulldown of NS2 was demonstrated upon blotting with anti-NS2 antibodies. Blotting with anti-ubiquitin antibodies again revealed a smear of bands (~50 to 250 kDa) in samples derived from HCV RNA-transfected cells following IP with anti-NS2 antibodies and not with IgG controls. No such signal appeared in samples derived from naive cells. Moreover, a ladder of bands with a similar molecular mass range was detected in HCV RNA-transfected samples upon blotting with anti-FLAG antibodies (albeit a single nonspecific band [~75 kDa] also appeared in samples derived from naive cells following IP with anti-NS2 antibodies). These results suggest that the NS2 polyubiquitination observed in the context of HCV infection was K63 linked.

To further characterize NS2 ubiquitination, Huh-7.5 cells were cotransfected with a plasmid encoding ubiquitin and either a GLuc-tagged NS2 or an empty GLuc plasmid. NS2 was immunoprecipitated from cell extracts using either anti-GLuc (Fig. 5E) or anti-NS2 (Fig. 5F) antibodies under denaturing conditions (42), followed by Western blotting with anti-ubiquitin antibodies. A smear of ubiquitin-conjugated bands (~35 to 55 kDa) was present in GLuc-NS2 samples pulled down with anti-GLuc antibodies but not the IgG control or in the empty plasmid sample (Fig. 5E). A similar smear (~35 to 80 kDa) was present in samples pulled down with anti-NS2 antibodies (Fig. 5F), indicating that these bands represent ubiquitin-conjugated NS2. Differences in the NS2 protein itself (WT versus GLuc tagged) and/or in the cellular milieu (infected versus naive) may explain the observed differences in the extent of polyubiquitination between our experimental systems. We predicted that the four lysine residues on the cytosolic surface of J6/JFH NS2, the highly conserved K27 and K173 and the less conserved K172 and K212 residues, would be ubiquitinated. We introduced single, triple (K27E-K172E-K173E; 3K), and quadruple (K27E-K172E-K173E-K212E; 4K) lysine-to-glutamic acid substitutions into the GLuc-NS2 vector and determined their effect on NS2 ubiquitination. Both the 3K and 4K NS2 mutations reduced the ubiquitin-conjugated bands to the background level, despite pulldown levels comparable to or greater than those seen with WT NS2 (Fig. 5E and F). In contrast, ubiquitination of the single-lysine NS2 mutants was unaffected or was reduced only slightly (Fig. 5E), suggesting that multiple lysine residues serve as the ubiquitin acceptors. None of the mutations impaired NS2 expression (Fig. 5G). Blotting with antibodies specific to polyubiquitin-K63 revealed a pattern similar to that observed with anti-pan-ubiquitin antibodies (Fig. 5E), further validating K63-linked polyubiquitination of NS2.

### NS2 K27 and K173 mediate HRS binding and HCV assembly.

Next, we studied the effect of NS2 lysine mutations on HRS binding by PCAs. The K27E, K173E, 4K, and, to a lesser extent, K172E NS2 mutations reduced HRS binding compared to WT NS2 results, whereas K212E had no significant effect (Fig. 5H). A similar effect on HRS binding was observed upon substitution of the lysine residues with arginine residues (see Fig. S5 in the supplemental material), suggesting that the phenotype observed with K-to-E mutations is unlikely to have resulted from an alteration in charge and/or structure. Together, these results suggest that HRS binding is mediated by lysine residues, particularly K27 and K173, within two distinct NS2 regions. To investigate the role of these lysine residues in the HCV life cycle, we introduced the single-lysine mutations into a bicistronic luciferase reporter HCV genome, J6/H77NS2/JFH(NS2-IRES-nsGluc2AUbi) (11), which enables expression of the HCV replicase independently of NS2-NS3 cleavage. While these mutations had no effect on HCV RNA replication (Fig. 5I), K27E and K173E severely impaired both intra- and extracellular infectivity relative to WT NS2 (Fig. 5J), consistent with an assembly defect. K27E and K173E severely impaired HCV assembly, in agreement with prior studies (16, 36). In accordance with their less pronounced effect on HRS binding, the K172E and K212E mutants showed moderate assembly defects and no apparent assembly defects, respectively. Together, these results suggest that polyubiquitination of NS2 K27, K172, and K173 may represent a mechanism underlying NS2’s roles in HRS binding and HCV assembly.

### Additional ESCRT components mediate HCV assembly, whereas Alix mediates viral release.

To define the roles of other ESCRT components, including additional hits identified in the screen, in infectious HCV production, we depleted CHMP1A, CHMP4B, VPS4A, VPS4B, or Alix by siRNAs with no effect on cellular viability (see Fig. S6A and B in the supplemental material). Similarly to HRS, depletion of CHMP1A, CHMP4B, VPS4A, or VPS4B had no effect on HCV RNA replication but significantly reduced both intra- and extracellular infectivity relative to NT control results, consistent with a defect in viral assembly (see Fig. S6C and D). In contrast, Alix was the only studied ESCRT component whose depletion increased intracellular infectivity while decreasing extracellular infectivity (see Fig. S6C and D). These results suggest that ESCRT-III and VPS/VTA1 proteins may collaborate with HRS in mediating HCV assembly, whereas Alix is essential for viral release.

## DISCUSSION

The ESCRT machinery has been previously implicated in infectious HCV production (19–21). Nevertheless, the precise role of the ESCRT machinery in this step of the HCV life cycle remained unclear. Moreover, the nature of HCV-ESCRT interactions remained unresolved since HCV structural proteins lack late domains associated with ESCRT recruitment. Here, we integrate proteomic, RNA interference (RNAi), viral genetic, and biophysical approaches to map the interaction network of HCV proteins with the ESCRT machinery and to test our hypothesis that HCV proteins recruit ESCRT components to mediate HCV envelopment. Our findings demonstrate HCV hijacking of HRS as an entry point into the ESCRT pathway to facilitate viral envelopment. Moreover, we demonstrate that NS2 undergoes ubiquitination and reveal a role for ubiquitinated NS2 residues in HRS recruitment and HCV assembly.

Nine novel interactions involving five HCV proteins and five ESCRT proteins were identified in our PCA screen. HRS was identified as a partner of several HCV proteins, and its interactions with NS2 and NS5A with the highest apparent affinity were validated in HCV-transfected cells. NS2 bound not only HRS but also the HRS regulator, SLC9A6 (29), and the latter was also bound by core. HRS has thus emerged as a critical entry point of HCV into the ESCRT network. This is in contrast to other viruses that generally access the ESCRT pathway by recruiting TSG101, Alix, or NEDD4 family proteins. It is tempting to speculate that by binding several HCV proteins, and particularly NS2, HRS concentrates viral particles at envelopment sites. Notably, HRS mediates multivalent interactions with ubiquitinated cargo by binding two ubiquitin molecules via its UIM (24) and forming a complex with STAM1/2 (44). Furthermore, HRS harbors PSAP and PPXY motifs, similarly to viral late domains, and its ectopic expression is sufficient to rescue the budding defect induced by HIV Gag PTAP mutations (45). HRS is therefore an ideal factor to provide access to the ESCRT network and compensate for the absence of defined late domains within HCV proteins.

**FIG 6:**
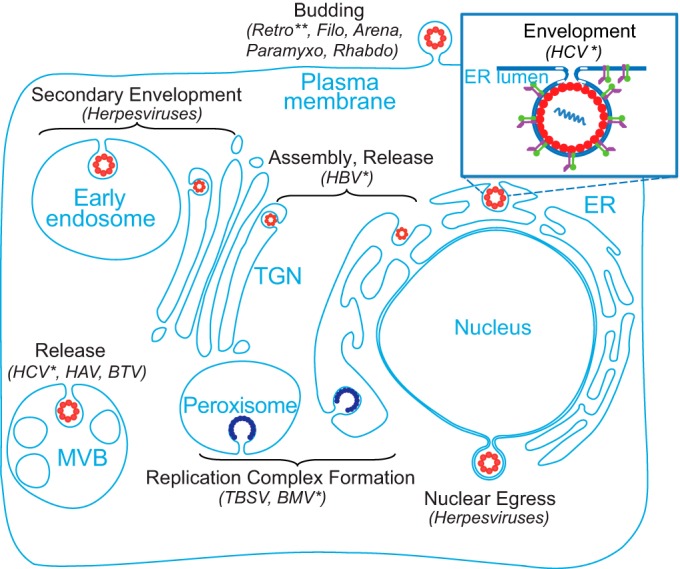
Model: roles of the ESCRT machinery in viral infections. The ESCRT machinery is hijacked by multiple DNA and RNA viruses to mediate various steps of the life cycle in distinct cellular compartments. The enlarged section (top right) highlights a novel role for the ESCRT machinery in HCV envelopment. The single asterisk (*) indicates a lack of a late domain. The paired asterisks (**) highlight the fact that foamy viruses are exceptional members of the *Retroviridae* that bud at the ER/TGN in addition to the plasma membrane. Nucleocapsids are indicated by red circles; replication complexes are indicated by dark blue semicircles; an HCV particle is indicated by a red circle with purple and green projections in the enlarged image. *Arena*, arenaviruses; *Filo*, filoviruses; *Paramyxo*, paramyxoviruses; *Retro*, retroviruses; *Rhabdo*, rhabdoviruses.

Our infectivity assays, core oligomerization assays, and biophysical analysis of intracellular viral particles revealed that HRS mediates HCV envelopment. These results thus define the precise role of ESCRT in infectious HCV production. The ESCRT machinery is hijacked by multiple viruses to mediate various steps of the viral life cycle in distinct cellular compartments, most commonly budding at the plasma membrane (Fig. 6). Unlike other *Retroviridae*, foamy viruses recruit the ESCRT machinery to facilitate budding at the ER and the trans-Golgi network (TGN), in addition to the plasma membrane (46). Nevertheless, with the exception of foamy viruses, to the best of our knowledge, the ESCRT machinery has not been previously implicated in envelopment of other RNA viruses that preferentially bud intracellularly. Moreover, all the viruses reported to date to hijack the ESCRT machinery to facilitate envelopment utilize late domains. Our results thus reveal a novel role for the ESCRT machinery in intracellular budding of an RNA virus lacking defined late domains.

We hypothesize that the noninfectious particles identified in HRS-depleted cells represent particles which acquired their envelope by budding into the ER but remained attached to the membrane via a “stalk” (similarly to budding-defective HIV particles harboring Gag late-domain mutations [45]). Since the ESCRT0 complex typically mediates cargo recognition, it is also possible that the incomplete envelopment results from a failure to recruit an essential cargo protein to the budding vesicle. Our attempts to distinguish between these possibilities by immunoelectron microscopy were, however, limited by the pleiomorphic shape of HCV particles and the low assembly efficiency; a known limitation in the HCV field (14). We predict that HCV proteins recruit ESCRT components to facilitate envelopment into the ER lumen. Indeed, the ESCRT was shown to be recruited to various cellular membrane structures, including ER-derived platforms (7, 8). We demonstrate localization of HRS to LD and ER in HCV-transfected cells. Contacts between endosomal and ER membranes may facilitate such recruitment (47).

Data indicate that NS2 brings together the viral structural and NS proteins, thereby orchestrating early and late HCV assembly steps (12, 16, 18). Our results suggest that NS2 and possibly core, P7, NS4A, and NS5A mediate HCV assembly in part by recruiting ESCRT proteins to facilitate envelopment. Moreover, our results indicate, for the first time, that NS2 is ubiquitinated both *in vitro* and in the context of HCV infection and suggest that recognition of ubiquitinated NS2 and possibly other viral proteins by HRS UIM may play a role in ESCRT recruitment and HCV assembly. The ubiquitinated NS2 residues thus appear to provide an alternative sorting signal to the ESCRT machinery in the absence of defined late domains. Future studies will determine whether ubiquitination plays a role in binding of HRS by NS5A, a protein previously shown to be ubiquitinated (34), and by P7 and NS4A. Interestingly, fusion of ubiquitin or ubiquitin ligases to retroviral Gag proteins is sufficient to drive viral budding, suggesting that ubiquitin can function as a late domain (2, 4). Nevertheless, ubiquitination acts redundantly with late domains in retrovirus infections and in other viral infections, providing a possible explanation of why the functional viral target of ubiquitination has not been identified in most cases. We thus reveal a novel role for K63 polyubiquitination of an NS protein in ESCRT recruitment and regulation of viral assembly and propose that ubiquitination functionally replaces a late domain in HCV infection. We cannot, however, exclude the possibility that a yet-to-be-defined viral late domain collaborates with this binding mechanism.

Our study results indicate that, in addition to HRS, components of the ESCRT-III and VPS4-VTA1 complexes are bound by core and NS5A, respectively, and that their depletion causes an assembly defect. We suggest that these ESCRT proteins collaborate with HRS to orchestrate HCV envelopment. Prior studies demonstrated impaired infectious HCV production following suppression of late ESCRT components (19–21). Nevertheless, data with respect to which precise step is mediated by ESCRT proteins appear to be conflicting, with the majority of data favoring a role in viral release. Moreover, these previous studies did not characterize the precise mechanism of ESCRT’s involvement in infectious HCV production. Our results thus, for the first time, comprehensively characterize the involvement of the ESCRT machinery in infectious HCV production. We believe that differences in experimental techniques (e.g., the more rigorous suppression of protein expression achieved in our study by the use of a validated siRNA sequence versus pooled siRNAs and by boosting at the time of electroporation) and/or viral backgrounds (J6/JFH here versus Jc-1 in prior studies) account for the discrepancy between some of those previous results and ours. Of the ESCRT proteins we studied, Alix was the only one dispensable for HCV assembly but essential for release. Since HCV directly recruits ESCRT-III proteins, Alix’s role in bridging the ESCRT-I and -III complexes (2) may be redundant in HCV assembly. We predict that Alix mediates HCV release by an ESCRT-independent mechanism such as trafficking to recycling endosomes (48).

Taken together, these results validate novel virus-host interactions required for HCV envelopment, reveal a novel role for ESCRT proteins in intracellular budding of a non-late-domain-containing virus, and suggest an entry mechanism into the ESCRT network in the absence of defined late domains. We speculate that these findings have potential implications for other RNA viruses and the design of novel antiviral strategies.

## MATERIALS AND METHODS

Plasmids, antibodies, RNAi, and primers are summarized in Text S1 in the supplemental material.

### RNAi.

Huh-7.5 cells were transduced with lentiviral particles harboring shRNAs (Sigma) or transfected with 200 nM single or pooled siRNAs by silMPORTER (Upstate/Millipore) and boosted with 50 nM siRNA after 48 h at the time of HCV transfection.

### Cell cultures.

Huh-7.5 cells and 293T cells were grown in Dulbecco’s modified Eagle’s medium (DMEM) supplemented with 10% fetal bovine serum (Omega Scientific), 1× nonessential amino acids, 1% l-glutamine, and 1% penicillin-streptomycin (Gibco).

### PCAs.

As previously described (22, 25), combinations of plasmids encoding prey (A) and bait (B) proteins, each fused to a fragment of the *Gaussia* luciferase protein (GLuc1 and GLuc2) or control vectors, were cotransfected into 293T cells plated in 96-well plates in triplicate. At 24 h posttransfection, cells were lysed and subjected to luciferase assays (Promega). Results were expressed as normalized luminescence ratios (NLR) calculated as follows: the average signal in cells transfected with GLuc1-A and GLuc2-B was divided by the average signal in wells transfected with GLuc1-A and an empty GLuc2 vector and those transfected with GLuc2-B and an empty GLuc1 vector.

### Coimmunoprecipitations.

Co-IPs in membrane fractions derived from HCV-transfected cells were carried out as described earlier (25) (see Text S1 in the supplemental material).

### IF confocal microscopy.

IF was performed in Huh-7.5 cells 72 h posttransfection with HCV RNA, as previously described (25).

### *In vitro* transcription of HCV RNA, electroporation, and viral titration.

These methods were performed as previously reported (25).

### HCV RNA replication and intra- and extracellular infectivity.

As previously described (25, 30), HCV RNA replication was measured by luciferase assays (Promega) in Huh-7.5 cell lysates 6 h and 72 h postelectroporation with HCV RNA harboring a luciferase reporter. At 72 h, electroporated cells were trypsinized, spun, resuspended in 500 µl medium, lysed by freeze-thaw cycles, and pelleted (5,000 × *g*, 10 min). To measure intra- and extracellular infectivity, these clarified lysates and culture supernatants, respectively, were used to inoculate naive cells in triplicate, followed by luciferase assays performed at 72 h.

### RNA extraction and qRT-PCR.

Total RNA was isolated from cells, cell culture supernatants, or gradient fractions by the use of a NucleoSpin RNA virus kit (Macherey-Nagel). HCV RNA was quantified by qRT-PCR, as previously described (25).

### Viability assays.

Cells were incubated for 2 h at 37°C with 10% alamarBlue reagent (TREK Diagnostic Systems). Fluorescence was detected by the use of a FlexStation II384 workstation.

### Core oligomerization assays.

HCV-transfected cells were lysed in a NativePAGE sample preparation kit (Invitrogen) with 1% *n*-dodecyl-β-d-maltoside under nonreducing conditions without heating and were loaded onto NativePAGE 4% to 16% Bis-Tris Gel (Invitrogen), as previously described (15). For two-dimensional resolution, individual lanes were cut out of the blue native PAGE gel, equilibrated in 2× SDS sample buffer for 30 min, stacked perpendicularly above a 12% polyacrylamide SDS gel in one large well, and overlaid with 1× SDS sample buffer. The well was sealed with 1% low-melting-temperature agarose prior to separation. Core protein was detected by Western blotting.

### Density gradient centrifugations.

Three days posttransfection with J6/JFH RNA, clarified cell lysates were loaded on continuous 10% to 60% sucrose gradients in TNE buffer (50 mM Tris-HCl [pH 7.4], 100 mM NaCl, 0.1 mM EDTA) and spun (16 h, 230,500 × *g*) at 4°C for isopycnic separation (32). For rate-zonal-like separation, lysates were layered on top of 0% to 30% sucrose density gradients in the presence or absence of 1% DDM and spun (1 h, 270,000 × *g*), as previously described (15). The refraction index of 10 to 13 collected fractions was measured by the use of a Milton-Roy refractometer.

To detect HCV proteins, ~200 µl of each fraction was incubated with 1% Triton for 30 min at 56°C. Volumes of 1 µl of heparin (1 mg/ml), 5.8 µl of NaCl (5 M), and 0.8 ml of methanol were then added, samples were subjected to vortex mixing, and proteins were precipitated by spinning for 5 min at 20,000 × *g*. Dried pellets were dissolved in 10 µl of 100 mM Tris buffer with 8 M urea and 40 µl of 5× SDS sample buffer.

### Proteolytic digestion protection assays.

As previously described (15), at 72 h following HCV transfection, cells seeded in 6-well plates were scraped into 250 µl of PK buffer (50 mM Tris-HCl [pH 8.0], 10 mM CaCl_2_, 1 mM dithiothreitol [DTT]) and subjected to freeze-thaw cycles. Samples (50 µl) were either left untreated or treated with 1% Triton X-100 for 5 min at room temperature and/or with 30, 50, or 200 µg/ml PK (Roche) for 1 h on ice. PK digestion was terminated by addition of 5 mM phenylmethylsulfonyl fluoride (PMSF) and 10 min of incubation on ice. The level of residual core was determined by immunoblotting.

### RNase digestion protection assays.

As previously described (15), 50-µl aliquots derived from gradient fraction no. 10 were (i) left untreated, (ii) treated with S7 RNase (Roche) (2 U; 30 min at 37°C), (iii) pretreated with PK (50 µg/ml in 10× PK buffer for 1 h on ice) followed by treatment with S7 RNase, or (iv) pretreated with 1% Triton X-100 (5 min at room temperature) prior to treatment with PK and S7 RNase. PK activity was stopped by adding 10 mM PMSF and protease inhibitors prior to S7 RNase digestion. Total RNA was extracted, and HCV RNA was quantified by qRT-PCR.

### NS2 protein expression and purification.

NS2 (92 to 216 aa)-GST fusion was expressed in *E. coli* C41 (DE3; Lucigen) and purified as previously described (40). Cultures were grown to an optical density at 600 (OD_600_) of 0.6, followed by induction with 0.1 mM IPTG (isopropyl-β-d-thiogalactopyranoside) for 18 h at 16°C. Cells were pelleted; lysed by 3 passes via the use of an EmulsiFlex-C5 homogenizer (Avestin) and a mixture consisting of 50 mM HEPES, 300 mM NaCl, 1 mM DTT, 5 mM MgCl_2_, 0.5% Triton, and protease inhibitors; and spun (48,000 × *g*, 20 min). Supernatants were incubated for 1 h with glutathione 4B-Sepharose beads at 4°C. After three washes, protein was eluted in a buffer containing 20 mM glutathione. The GST tag was cleaved by thrombin (GE Healthcare).

### *In vitro* ubiquitination assay.

A 5-µg volume of recombinant NS2 was incubated with Huh-7.5 cell extract (24 µg total protein), 2 µM ubiquitin–aldehyde, 0.5 µg/µl ubiquitin, 10 µM MG-132, and components of a ubiquitination kit (Enzo), including E1 and E2 (UBCH2) enzymes and Mg-ATP, in a final volume of 20 µl. After 20 to 60 min of incubation at 37°C, the reaction was stopped by addition of SDS sample buffer. Samples were separated by SDS-PAGE.

### Detection of ubiquitination by IP (42).

GLuc-NS2 or HCV (Jc1)-transfected and control cells were treated for 2 h with 10 µM MG-132 and lysed in a buffer containing 8 M urea (see Text S1 in the supplemental material). Lysates were diluted to 4 M urea in IP buffer and spun (14,000 rpm, 10 min). Clarified supernatants were precleared with protein A/G Dynabeads and incubated with anti-GLuc, anti-NS2, or IgG antibodies. Beads capturing the antibodies were washed and resuspended in SDS.

### Detection of ubiquitination using anti-K63 TUBE technology.

HCV (Jc1)-transfected and control cells were treated for 3 h with 10 µM MG-132, washed twice with cold phosphate-buffered saline (PBS), and lysed in lysis buffer containing 500 nM FLAG anti-K63 TUBE reagent (LifeSensors), 100 mM Tris-HCl (pH 8.0), 0.15 M NaCl, 5 mM EDTA, 1% NP-40, 0.5% Triton-X 100, deubiquitinase (DUB) inhibitors (100 µM PR619, 5 mM 1,10-phenanthroline [o-PA], 5 mM *N*-ethylmaleimide [NEM; LifeSensors]), and a protease inhibitor cocktail. In accordance with the instructions of the manufacturers, clarified lysates were resuspended in reaction buffer containing 500 nM FLAG anti-K63 TUBE reagent and incubated for 1 h on ice. A 5-µg volume of anti-NS2 or IgG antibodies was then added followed by 16 h of incubation at 4°C. Next, samples were incubated for 2 h with A/G Dynabeads, followed by extensive washing with Catch and Release IP wash buffer (EMD Millipore) supplemented with 4 M urea and elution with 5× SDS sample buffer and boiling.

### Statistical analysis.

To normalize the PCA screen data, we fitted a Gaussian function to the distribution of RRS measurements. A *z* score was calculated for each interaction following a logarithmic transformation by subtracting the mean value of this fit from the interaction signal and dividing the resulting value by the SD of the fit. *P* values were calculated using a two-tailed unpaired Student’s *t* test.

### Accession number(s).

The accession numbers for the sequences used in this work are provided in Tables S1 to S3 in the supplemental material.

## SUPPLEMENTAL MATERIAL

Text S1  Supplemental Materials and Methods. Text S1 provides lists of the plasmids, antibodies, and primers used in this study. Moreover, it provides a more detailed description of the co-IP experiments and detection of ubiquitination by IP. Download Text S1, PDF file, 0.3 MB

Figure S1  Expression of the viral proteins used in the PCA screen. 293T cells were transfected with plasmids encoding individual viral proteins fused to a GLuc2 tag or an empty GLuc2 plasmid. For NS3/NS4A coexpression (right panels), cells were cotransfected with GLuc2-NS3- and FLAG-NS4A-encoding plasmids or with the corresponding empty plasmids. Cell lysates collected at 24 h posttransfection were subjected to Western blot analysis using anti-GLuc, anti-FLAG, and anti-actin antibodies. Molecular mass marker mobility data are shown at the left in kilodaltons. Left panel: longer exposure of the control and P7 lanes. Download Figure S1, PDF file, 0.6 MB

Figure S2  HRS is recruited to LDs and the ER to mediate HCV assembly. (A) HRS/S18 RNA ratio measured by qRT-PCR in Huh-7.5 cells transfected with a pool of four siRNAs (ON-TARGETplus SMARTpools; Dharmacon) targeting HRS or a pool of nontargeting (NT) sequences at 48 h posttransfection. (B) HRS protein levels determined by quantitative Western blotting in cells at 48 h posttransfection with the corresponding pooled siRNAs. Numbers represent HRS-to-actin protein ratios relative to the NT control. (C) Cellular viability determined by alamarBlue assays at 48 h posttransfection with siRNAs. Plotted data represent relative fluorescence values normalized to the NT control. (D) Cells were electroporated with J6/JFH(p7-Rluc2A) at 48 h posttransfection with the indicated pooled siRNAs. HCV RNA replication in these cells was determined by luciferase assays at 6 h (black) and 72 h (white) postelectroporation. (E) Intracellular (black) and extracellular (white) infectivity measured in naive Huh-7.5 cells infected with clarified cell lysates and supernatants derived from electroporated cells harboring the indicated siRNAs by luciferase assays, respectively. (F) Infectious virus production measured by limiting dilution assays*.* TCID_50_, 50% tissue culture infectious dose. (G and H) Representative images of HRS (red) and the LD marker Bodipy (G) or the ER marker calnexin (green) (H) in naive and HCV-transfected cells. Graphs represent percent colocalization (M2 values) of the indicated signals averaged from at least 20 cells for each category. Means ± SD (error bars) of results from at least two independent experiments are shown. RLU, relative light units. *, *P* < 0.05; **, *P* < 0.01; ***, *P* < 0.001 (Student’s *t* test). Download Figure S2, PDF file, 2.5 MB

Figure S3  HRS depletion does not alter core cosedimentation with the HCV RNA. Clarified cell lysates derived from HCV-transfected HRS-depleted or control NT cell lines were layered on top of a continuous sucrose gradient (10% to 60%) and spun for 16 h at 36,000 rpm. A total of 13 fractions were collected and subjected to measurement of buoyant density by the use of a refractometer and of HCV RNA levels by qRT-PCR. Plotted data represent the buoyant density along the gradient (right axis) and the HCV RNA copy number per fraction (left axis). Download Figure S3, PDF file, 0.2 MB

Figure S4  NS2 undergoes ubiquitination *in vitro* and in HCV-transfected cells. (A to C) Expression and purification of recombinant NS2. (A) Truncated NS2 protein (92 to 216 aa), fused to a C-terminal GST tag, was expressed in *E. coli* and purified on glutathione beads. Samples (5 µg) of protein from six elution fractions (E1 to E6) were separated by SDS-PAGE. NS2-GST was detected by immunoblotting with anti-NS2 antibodies. Following cleavage of the GST tag, protein samples (0.5 µg to 20 µg) were separated by SDS-PAGE. (B and C) Membranes were blotted with anti-NS2 (B) and anti-GST (C) antibodies. An ~14-kDa band corresponding to GST-cleaved truncated NS2 protein is shown in panel B. (D) Lysates of HCV RNA-transfected or naive Huh-7.5 cells were subjected to IP with anti-NS2 antibodies or IgG under denaturing conditions. Representative membranes blotted with anti-NS2 and anti-ubiquitin (UB) antibodies are shown. Download Figure S4, PDF file, 0.5 MB

Figure S5  NS2 K-to-R mutations reduce HRS binding. (A) Levels of NS2 in lysates of cells transfected with the indicated plasmids. (B and C) Analysis of interactions of WT NS2 (B) or mutant NS2 with HRS (C) by PCAs. Plotted data represent NLRs relative to WT NS2-HRS binding. Mean values and standard deviations of results of 2 experiments (each performed in quadruplicate) are shown. **, *P* < 0.01 (Student’s *t* test). Download Figure S5, PDF file, 0.5 MB

Figure S6  Additional ESCRT components mediate HCV assembly, whereas Alix mediates viral release. Huh-7.5 cells were transfected with the indicated siRNAs. (A) Representative membranes showing protein expression at 48 h posttransfection. (B) Cellular viability measured by alamarBlue-based assays and expressed as fluorescence values normalized to an NT control. (C) HCV RNA replication 6 and 72 h postelectroporation with *in vitro* transcribed HCV RNA measured by luciferase assays. (D) Intra- and extracellular infectivity by luciferase assays in naive cells inoculated with clarified cell lysates and supernatants derived from the HCV-electroporated cells, respectively. Plotted data represent infectivity normalized to NT controls. Mean values ± SD of results of 3 independent experiments are shown. **, *P* < 0.01; ***, *P* < 0.001 (Student’s *t* test). Download Figure S6, PDF file, 0.3 MB

Table S1  Analysis of ESCRT-HCV interactions by PCAs. The first and second columns list gene symbols and accession numbers. Numbers in parentheses represent several copies of the same open reading frame (ORF) in the human ORFeome library. The second to eighth columns list the average *z* scores measured by PCAs in three independent experiments each in triplicate. Table S1, PDF file, 0.1 MB

Table S2  Protein interactions of a random reference set (RRS) tested by PCAs to benchmark the screen. The first four columns list gene symbols and accession numbers of the interactors (A and B). The fifth column lists the average *z* scores measured by PCAs in three independent experiments each in triplicate. Table S2, PDF file, 0.2 MB

Table S3  Known positive protein interactions tested by PCAs. The first four columns list gene symbols and accession numbers of the interactors (A and B). The fifth column lists the average *z* scores measured by PCAs in three independent experiments each in triplicate. Table S3, PDF file, 0.1 MB

## References

[1] Hurley JH, Hanson PI 2010 Membrane budding and scission by the ESCRT machinery: it’s all in the neck. Nat Rev Mol Cell Biol 11:556–566. doi:10.1038/nrm2937. 20588296 PMC2922035

[2] Votteler J, Sundquist WI 2013 Virus budding and the ESCRT pathway. Cell Host Microbe 14:232–241. doi:10.1016/j.chom.2013.08.012. 24034610 PMC3819203

[3] Nathan JA, Kim HT, Ting L, Gygi SP, Goldberg AL 2013 Why do cellular proteins linked to K63-polyubiquitin chains not associate with proteasomes? EMBO J 32:552–565. doi:10.1038/emboj.2012.354. 23314748 PMC3579138

[4] Shields SB, Piper RC 2011 How ubiquitin FUNCTIONS with ESCRTS. Traffic 12:1306–1317. doi:10.1111/j.1600-0854.2011.01242.x. 21722280 PMC3171646

[5] Feng Z, Hensley L, McKnight KL, Hu F, Madden V, Ping L, Jeong S-H, Walker C, Lanford RE, Lemon SM 2013 A pathogenic picornavirus acquires an envelope by hijacking cellular membranes. Nature 496:367–371. doi:10.1038/nature12029. 23542590 PMC3631468

[6] Wirblich C, Bhattacharya B, Roy P 2006 Nonstructural protein 3 of bluetongue virus assists virus release by recruiting ESCRT-I protein Tsg101. J Virol 80:460–473. doi:10.1128/JVI.80.1.460-473.2006. 16352570 PMC1317520

[7] Barajas D, Jiang Y, Nagy PD 2009 A unique role for the host ESCRT proteins in replication of tomato bushy stunt virus. PLoS Pathog 5:e1000705. doi:10.1371/journal.ppat.1000705. 20041173 PMC2791863

[8] Diaz A, Zhang J, Ollwerther A, Wang X, Ahlquist P 2015 Host ESCRT proteins are required for bromovirus RNA replication compartment assembly and function. PLoS Pathog 11:e1004742. doi:10.1371/journal.ppat.1004742. 25748299 PMC4351987

[9] Welsch S, Müller B, Kräusslich H-G 2007 More than one door—budding of enveloped viruses through cellular membranes. FEBS Lett 581:2089–2097. doi:10.1016/j.febslet.2007.03.060. 17434167 PMC7126970

[10] Watanabe T, Sorensen EM, Naito A, Schott M, Kim S, Ahlquist P 2007 Involvement of host cellular multivesicular body functions in hepatitis B virus budding. Proc Natl Acad Sci U S A 104:10205–10210. doi:10.1073/pnas.0704000104. 17551004 PMC1891263

[11] Jones CT, Murray CL, Eastman DK, Tassello J, Rice CM 2007 Hepatitis C virus p7 and NS2 proteins are essential for production of infectious virus. J Virol 81:8374–8383. doi:10.1128/JVI.00690-07. 17537845 PMC1951341

[12] Jirasko V, Montserret R, Lee JY, Gouttenoire J, Moradpour D, Penin F, Bartenschlager R 2010 Structural and functional studies of nonstructural protein 2 of the hepatitis C virus reveal its key role as organizer of virion assembly. PLoS Pathog 6:e1001233. doi:10.1371/journal.ppat.1001233. 21187906 PMC3002993

[13] Steinmann E, Penin F, Kallis S, Patel AH, Bartenschlager R, Pietschmann T 2007 Hepatitis C virus p7 protein is crucial for assembly and release of infectious virions. PLoS Pathog 3:e103. doi:10.1371/journal.ppat.0030103. 17658949 PMC1924870

[14] Bartenschlager R, Penin F, Lohmann V, André P 2011 Assembly of infectious hepatitis C virus particles. Trends Microbiol 19:95–103. doi:10.1016/j.tim.2010.11.005. 21146993

[15] Gentzsch J, Brohm C, Steinmann E, Friesland M, Menzel N, Vieyres G, Perin PM, Frentzen A, Kaderali L, Pietschmann T 2013 Hepatitis C virus p7 is critical for capsid assembly and envelopment. PLoS Pathog 9:e1003355. doi:10.1371/journal.ppat.1003355. 23658526 PMC3642076

[16] Phan T, Beran RK, Peters C, Lorenz IC, Lindenbach BD 2009 Hepatitis C virus NS2 protein contributes to virus particle assembly via opposing epistatic interactions with the E1-E2 glycoprotein and NS3-NS4A enzyme complexes. J Virol 83:8379–8395. doi:10.1128/JVI.00891-09. 19515772 PMC2738163

[17] Dentzer TG, Lorenz IC, Evans MJ, Rice CM 2009 Determinants of the hepatitis C virus nonstructural protein 2 protease domain required for production of infectious virus. J Virol 83:12702–12713. doi:10.1128/JVI.01184-09. 19812162 PMC2786863

[18] Popescu CI, Callens N, Trinel D, Roingeard P, Moradpour D, Descamps V, Duverlie G, Penin F, Héliot L, Rouillé Y, Dubuisson J 2011 NS2 protein of hepatitis C virus interacts with structural and non-structural proteins towards virus assembly. PLoS Pathog 7:e1001278. doi:10.1371/journal.ppat.1001278. 21347350 PMC3037360

[19] Corless L, Crump CM, Griffin SD, Harris M 2010 Vps4 and the ESCRT-III complex are required for the release of infectious hepatitis C virus particles. J Gen Virol 91:362–372. doi:10.1099/vir.0.017285-0. 19828764 PMC7615705

[20] Ariumi Y, Kuroki M, Maki M, Ikeda M, Dansako H, Wakita T, Kato N 2011 The ESCRT system is required for hepatitis C virus production. PLoS One 6:e14517. doi:10.1371/journal.pone.0014517. 21264300 PMC3019154

[21] Tamai K, Shiina M, Tanaka N, Nakano T, Yamamoto A, Kondo Y, Kakazu E, Inoue J, Fukushima K, Sano K, Ueno Y, Shimosegawa T, Sugamura K 2012 Regulation of hepatitis C virus secretion by the Hrs-dependent exosomal pathway. Virology 422:377–385. doi:10.1016/j.virol.2011.11.009. 22138215

[22] Cassonnet P, Rolloy C, Neveu G, Vidalain P, Chantier T, Pellet J, Jones L, Muller M, Demeret C, Gaud G, Vuillier F, Lotteau V, Tangy F, Favre M, Jacob Y 2011 Benchmarking a luciferase complementation assay for detecting protein complexes. Nat Methods 8:990–992. doi:10.1038/nmeth.1773. 22127214

[23] Op De Beeck A, Cocquerel L, Dubuisson J 2001 Biogenesis of hepatitis C virus envelope glycoproteins. J Gen Virol 82:2589–2595. doi:10.1099/0022-1317-82-11-2589. 11602769

[24] Hirano S, Kawasaki M, Ura H, Kato R, Raiborg C, Stenmark H, Wakatsuki S 2006 Double-sided ubiquitin binding of Hrs-UIM in endosomal protein sorting. Nat Struct Mol Biol 13:272–277. doi:10.1038/nsmb1051. 16462748

[25] Neveu G, Barouch-Bentov R, Ziv-Av A, Gerber D, Jacob Y, Einav S 2012 Identification and targeting of an interaction between a tyrosine motif within hepatitis C virus core protein and AP2M1 essential for viral assembly. PLoS Pathog 8:e1002845. doi:10.1371/journal.ppat.1002845. 22916011 PMC3420927

[26] Rual JF, Hirozane-Kishikawa T, Hao T, Bertin N, Li S, Dricot A, Li N, Rosenberg J, Lamesch P, Vidalain PO, Clingingsmith TR, Hartley JL, Esposito D, Cheo D, Moore T, Simmons B, Sequerra R, Bosak S, Doucette-Stamm L, Le Peuch C, Vandenhaute J, Cusick M, Albala J, Hill D, Vidal M 2004 Human ORFeome version 1.1: a platform for reverse proteomics. Genome Res 14:2128–2135. doi:10.1101/gr.2973604. 15489335 PMC528929

[27] Lindenbach BD, Evans MJ, Syder AJ, Wölk B, Tellinghuisen TL, Liu CC, Maruyama T, Hynes RO, Burton DR, McKeating JA, Rice CM 2005 Complete replication of hepatitis C virus in cell culture. Science 309:623–626. doi:10.1126/science.1114016. 15947137

[28] Lin C, Thomson JA, Rice CM 1995 A central region in the hepatitis C virus NS4A protein allows formation of an active NS3-NS4A serine proteinase complex in vivo and in vitro. J Virol 69:4373–4380. 7769699 10.1128/jvi.69.7.4373-4380.1995 PMC189178

[29] Mitsui K, Koshimura Y, Yoshikawa Y, Matsushita M, Kanazawa H 2011 The endosomal Na+/H+ exchanger contributes to multivesicular body formation by regulating the recruitment of ESCRT-0 Vps27p to the endosomal membrane. J Biol Chem 286:37625–37638. doi:10.1074/jbc.M111.260612. 21896492 PMC3199507

[30] Murray CL, Jones CT, Tassello J, Rice CM 2007 Alanine scanning of the hepatitis C virus core protein reveals numerous residues essential for production of infectious virus. J Virol 81:10220–10231. doi:10.1128/JVI.00793-07. 17634240 PMC2045476

[31] Schägger H, Cramer WA, von Jagow G 1994 Analysis of molecular masses and oligomeric states of protein complexes by blue native electrophoresis and isolation of membrane protein complexes by two-dimensional native electrophoresis. Anal Biochem 217:220–230. doi:10.1006/abio.1994.1112. 8203750

[32] Gastaminza P, Kapadia SB, Chisari FV 2006 Differential biophysical properties of infectious intracellular and secreted hepatitis C virus particles. J Virol 80:11074–11081. doi:10.1128/JVI.01150-06. 16956946 PMC1642172

[33] Suzuki R, Moriishi K, Fukuda K, Shirakura M, Ishii K, Shoji I, Wakita T, Miyamura T, Matsuura Y, Suzuki T 2009 Proteasomal turnover of hepatitis C virus core protein is regulated by two distinct mechanisms: a ubiquitin-dependent mechanism and a ubiquitin-independent but PA28γ-dependent mechanism. J Virol 83:2389–2392. doi:10.1128/JVI.01690-08. 19091860 PMC2643730

[34] Hou W, Tian Q, Zheng J, Bonkovsky HL 2010 Zinc mesoporphyrin induces rapid proteasomal degradation of hepatitis C nonstructural 5A protein in human hepatoma cells. Gastroenterology 138:1909–1919. doi:10.1053/j.gastro.2009.11.001. 19909748 PMC2860067

[35] Saeed M, Suzuki R, Watanabe N, Masaki T, Tomonaga M, Muhammad A, Kato T, Matsuura Y, Watanabe H, Wakita T, Suzuki T 2011 Role of the endoplasmic reticulum-associated degradation (ERAD) pathway in degradation of hepatitis C virus envelope proteins and production of virus particles. J Biol Chem 286:37264–37273. doi:10.1074/jbc.M111.259085. 21878646 PMC3199473

[36] De la Fuente C, Goodman Z, Rice CM 2013 Genetic and functional characterization of the N-terminal region of the hepatitis C virus NS2 protein. J Virol 87:4130–4145. doi:10.1128/JVI.03174-12. 23408609 PMC3624385

[37] Welbourn S, Jirasko V, Breton V, Reiss S, Penin F, Bartenschlager R, Pause A 2009 Investigation of a role for lysine residues in non-structural proteins 2 and 2/3 of the hepatitis C virus for their degradation and virus assembly. J Gen Virol 90:1071–1080. doi:10.1099/vir.0.009944-0. 19264595

[38] Thibeault D, Maurice R, Pilote L, Lamarre D, Pause A 2001 In vitro characterization of a purified NS2/3 protease variant of hepatitis C virus. J Biol Chem 276:46678–46684. doi:10.1074/jbc.M108266200. 11591719

[39] Lorenz IC, Marcotrigiano J, Dentzer TG, Rice CM 2006 Structure of the catalytic domain of the hepatitis C virus NS2−3 protease. Nature 442:831–835. doi:10.1038/nature04975. 16862121

[40] Lu A, Pfeffer SR 2013 Golgi-associated RhoBTB3 targets cyclin E for ubiquitylation and promotes cell cycle progression. J Cell Biol 203:233–250. doi:10.1083/jcb.201305158. 24145166 PMC3812982

[41] Pietschmann T, Kaul A, Koutsoudakis G, Shavinskaya A, Kallis S, Steinmann E, Abid K, Negro F, Dreux M, Cosset FL, Bartenschlager R 2006 Construction and characterization of infectious intragenotypic and intergenotypic hepatitis C virus chimeras. Proc Natl Acad Sci U S A 103:7408–7413. doi:10.1073/pnas.0504877103. 16651538 PMC1455439

[42] Newton K, Matsumoto ML, Ferrando RE, Wickliffe KE, Rape M, Kelley RF, Dixit VM 2012 Using linkage-specific monoclonal antibodies to analyze cellular ubiquitylation, p–196 *In* Dohmen RJ, Scheffner M (ed), Ubiquitin family modifiers and the proteasome, vol. 832. Humana Press, New York, NY. 10.1007/978-1-61779-474-2_13 22350886

[43] Sims JJ, Scavone F, Cooper EM, Kane LA, Youle RJ, Boeke JD, Cohen RE 2012 Polyubiquitin-sensor proteins reveal localization and linkage-type dependence of cellular ubiquitin signaling. Nat Methods 9:303–309. doi:10.1038/nmeth.1888. 22306808 PMC3438894

[44] Mayers JR, Fyfe I, Schuh AL, Chapman ER, Edwardson JM, Audhya A 2011 ESCRT-0 assembles as a heterotetrameric complex on membranes and binds multiple ubiquitinylated cargoes simultaneously. J Biol Chem 286:9636–9645. doi:10.1074/jbc.M110.185363. 21193406 PMC3058970

[45] Pornillos O, Higginson DS, Stray KM, Fisher RD, Garrus JE, Payne M, He G-P, Wang HE, Morham SG, Sundquist WI 2003 HIV gag mimics the Tsg101-recruiting activity of the human Hrs protein. J Cell Biol 162:425–434. doi:10.1083/jcb.200302138. 12900394 PMC2172688

[46] Hütter S, Zurnic I, Lindemann D 2013 Foamy virus budding and release. Viruses 5:1075–1098. doi:10.3390/v5041075. 23575110 PMC3705266

[47] Eden ER, White IJ, Tsapara A, Futter CE 2010 Membrane contacts between endosomes and ER provide sites for PTP1B-epidermal growth factor receptor interaction. Nat Cell Biol 12:267–272. doi:10.1038/ncb2026. 20118922

[48] Shi A, Pant S, Balklava Z, Chen CC, Figueroa V, Grant BD 2007 A novel requirement for C. elegans Alix/ALX-1 in RME-1 mediated membrane transport. Curr Biol 17:1913–1924. doi:10.1016/j.cub.2007.10.045. 17997305 PMC2175126

