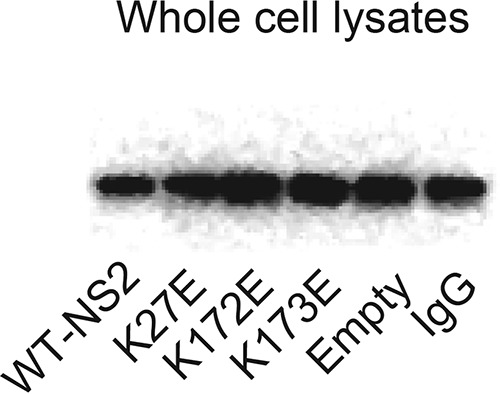# Erratum for Barouch-Bentov et al., “Hepatitis C Virus Proteins Interact with the Endosomal Sorting Complex Required for Transport (ESCRT) Machinery via Ubiquitination To Facilitate Viral Envelopment”

**DOI:** 10.1128/mBio.02234-17

**Published:** 2018-01-09

**Authors:** Rina Barouch-Bentov, Gregory Neveu, Fei Xiao, Melanie Beer, Elena Bekerman, Stanford Schor, Joseph Campbell, Jim Boonyaratanakornkit, Brett Lindenbach, Albert Lu, Yves Jacob, Shirit Einav

**Affiliations:** a Division of Infectious Diseases and Geographic Medicine, Department of Medicine, and Department of Microbiology and Immunology, Stanford University School of Medicine, Stanford, California, USA; b Department of Microbial Pathogenesis, Yale School of Medicine, New Haven, Connecticut, USA; c Department of Biochemistry, Stanford University School of Medicine, Stanford, California, USA; d Département de Virologie, Unité de Génétique Moléculaire des Virus ARN (GMVR), Institut Pasteur, Centre national de la recherche scientifique, and Université Paris Diderot, Paris, France

## ERRATUM

Volume 7, no. 6, e01456-16, 2016, https://doi.org/10.1128/mBio.01456-16. The bottom middle panel of Fig. 5E has now been changed. The original membrane shown in this panel was missing the sample of the whole-cell lysate for the IgG control, as the membrane was cropped without it by mistake. As shown, the level of actin in the IgG sample was comparable to that of the other samples, and therefore, the interpretation of the data is unchanged.

**Figure 1:**