# Supplementary material for: Hepatitis C Virus Proteins Interact with the Endosomal Sorting Complex Required for Transport (ESCRT) Machinery via Ubiquitination To Facilitate Viral Envelopment
Source: mBio. 2016 Nov 1;7(6):e01456-16. doi: 10.1128/mBio.01456-16 (PMC5090039; doi:10.1128/mBio.01456-16)
Supplement: Text S1 — Supplemental Materials and Methods. Text S1 provides lists of the plasmids, antibodies, and primers used in this study. Moreover, it provides a more detailed description of the co-IP experiments and detection of ubiquitination by IP. Download [file mbo005163053s1.pdf]

Text S1

Supplemental Materials and Methods

Plasmids. ORFs encoding host proteins were selected from the Human ORFeome library (1) (Open biosystems) and recombined into pGLuc vector (for luciferase tagging) (2) by gateway technology (Invitrogen). ORFs encoding HCV proteins were amplified from described vectors (3) and recombined into pGLuc or pFLAG vectors. pFL-J6/JFH(p7-Rluc2A) (3), J6/H77NS2/JFH(NS2-IRES-nsGluc2AUbi)(4) and J6/H77NS2/JFH (5) were a gift from Dr. Rice. Point mutations were introduced by the QuikChange kit (Stratagene). Plasmid encoding the HCV JC1 genome was previously described (6). A truncated cytoplasmic NS2 (NS2 92-216 aa) was cloned from J6/JFH and ligated into Pgex-4T1 plasmid (Addgene) for fusion of a C-terminal GST tag.

Antibodies. Rabbit anti-Alix, rabbit anti-HRS, mouse anti-ubiquitin (P4D1), mouse anti-GST (B-14), and normal mouse and rabbit IgG were from Santa Cruz Biotechnology. Rabbit anti-CHMP1A, rabbit anti-CHMP4B, rabbit anti-VPS4A or VPS4B, mouse anti-calnexin (anti-CANX) and mouse anti-actin antibodies were from Sigma. Rabbit anti-HRS antibodies for IP (A300-989A) were from Bethyl. Mouse anti-core (C7-50) antibodies were from Abcam and mouse anti-core (6G7, Ascites) from Virostat. Mouse anti-ubiquitin antibodies (Lys63-Specific) were from Millipore. Rabbit anti-GLuc antibodies were from NEB BioLabs. Rabbit anti-NS2 antibodies were a gift from Dr. Lindenbach. Mouse anti-NS2(6H6) and mouse anti-NS5A antibodies were a gift from Dr. Rice. Anti-rabbit Alexa Fluor 488 and goat anti-mouse Alexa Fluor 594 antibodies were from Invitrogen.

RNAi. MISSION Lentiviral Transduction Particles targeting HRS (TRCN 609 and 897) were from Sigma. On-TARGETplus SMARTpools targeting HRS (LQ-008170-00-0002, J-004233-05, J-004233-06, J-004233-07, and J-004233-08), Alix (J-004233-09, J-004233-10, J-004233-11, J-004233-12), and a non-targeting control (D-001810-10-05) were from Dharmacon. siRNAs targeting CHMP1A (S10142), CHMP4B (S43362), VPS4A (20167), VPS4B (15440), and a non-targeting control (4390843) were from Life Technologies.

Primers sequence.
1. HRS A266Q/A268Q-FWD:
AGGAGGAGGAGCTGCAGCTGCAGCTGCAGCTGTCACAGTCAGAGGCGGA
2. HRS L269A/S270A -FWD:
TGCAGCTGGCCCTGGCGGCGGCACAGTCAGAGGCGGAGGA
3. NS2 K27E-FWD:
CTTTACTCTCACCCCCGGGTATGAGACCCTTCTCAGCCGGTTTTTG
4. NS2 K172E-FWD: TCATCTTCAGTCCGATGGAGGAGAAAGTCATTGTCTGGGGAGC
5. NS2 K173E-FWD: TCTTCAGTCCGATGGAGAAGGAAGTCATTGTCTGGGGAGCGGA
6. NS2 K212E (for the GLuc-NS2 plasmid)-FWD:
CAGCTGATGGCTATACCTCCGAGGGGTGGAGTCTTCTCTAATA
7. NS2 K212E (for the Bicistronic H77NS2/JFH plasmid)-FWD:
CAGCTGATGGCTATACCTCCGAGGGGTGGAGTCTTC
8. NS2 (92-216 aa)-GST-FWD: GTTCCGCGTGGATCC CCTAAAAGGTGCTTTGACGC
9. NS2 (92-216 aa)-GST-REV: GCAGATCGTCAGTCAGAGAAGACTCCACCCCTTGG

Co-immunoprecipitations.
Membranes were prepared from ~20×10^6 Huh-7.5 cells transfected with HCV J6/JFH or J6/H77NS2/JFH (5) 3 days posttransfection, as we have previously described (7). Briefly, cells were collected by trypsinization, washed once with PBS and incubated with 1 mM dithiobis-succinimidyl-propionate (DSP) cross-linker (Pierce) solution to allow covalent binding of the already bound interacting proteins. Samples were incubated for 2 hr on ice. Tris (pH 7.5) was added up to 200 mM to quench unreacted DSP. Cells were washed once with PBS, resuspended in HME buffer (20 mM HEPES [pH 7.4], 1 mM EDTA, 2 mM MgCl2), supplemented with phenylmethylsulfonyl fluoride to a final concentration of 1 mM and a protease inhibitors cocktail (Sigma). Cells were lysed by two cycles of freeze-thaw in dry ice-ethanol and then passaged through a 27.5-gauge needle 10 times. Nuclei were removed by centrifugation at 250×g for 10 min, and the postnuclear supernatant was subjected to ultracentrifugation at 100,000×g for 30 min to obtain the membrane preparation. All steps were done at 4 °C. Membrane pellets were resuspended in 100 µl HME buffer (20 mM NaHEpes (pH 7.4), 1 mM EDTA (pH 8), 2 mM MgCl2). TDB buffer (2.5% Triton X-100, 25 mM triethanolamine-Cl [pH 8.6], 20 mM NaCl, 5 mM EDTA, 0.2% NaN3) was added for a final volume of 1 ml. Samples were incubated overnight with either anti-HRS antibodies, anti-NS2, anti-NS5A antibodies or IgG controls, and protein G magnetic beads (Dynabeads, Life Technologies). Following extensive washes of beads, bound proteins were eluted by adding SDS sample buffer and heating for 10 min. Immunoprecipitates were analyzed by Western blotting. Experiments were conducted twice.

Detection of ubiquitination by IP (8). GLuc-NS2 or HCV (Jc1) transfected cells and control cells were treated for 3 hr with 10 µM MG-132 24hr or 72hr posttransfection, respectively. Cells were then lysed in 8 M Urea, 20 mM Tris/pH7.5, 135 mM NaCl, 1% Triton X-100, 10% glycerol, 1.5 mM MgCl2, 5mM EDTA, and 15mM N-ethylmalemide for 30 min at RT. Lysates were diluted to 4 M Urea in IP buffer and spun (14,000 RPM, 10 min). Clarified supernatants were precleared with 100 µL Protein A/G Dynabeads for 1hr followed by overnight incubation with anti-GLuc, anti-NS2 or IgG antibodies. Antibodies were captured with 100 µL beads for 30 min at RT. Beads were extensively washed with buffer and resuspended in 80 µL Catch and Release denaturing elution buffer (MILLIPORE). Immunoprecipitates were eluted by heating and analyzed by Western blotting.

Supplemental References

1. **Rual JF, Hirozane Kishikawa T, Hao T, Bertin N, Li S, Dricot A, Li N, Rosenberg J, Lamesch P, Vidalain PO, Clingingsmith T, Hartley J, Esposito D, Cheo D, Moore T, Simmons B, Sequerra R, Bosak S, Doucette-Stamm L, Le Peuch C, Vandenhaute J, Cusick M, Albala J, Hill D, Vidal M.** 2004. Human ORFeome version 1.1: a platform for reverse proteomics. Genome research **14:**2128-2135.
2. **Cassonnet P, Rolloy, C., Neveu, G., Vidalain, P.O., Chnatier, T., Pellet, J., Jones,L., Muller, M., Demeret, C., Gaud, G., Vuillier, F., Lotteau, V., Tangy, F., Favre, M., Jacob, Y.** 2011. Benchmarking a luciferase complementation assay for detecting protein complexes. Nat Methods **8:**990-992.
3. **Murray CL, Jones CT, Tassello J, Rice CM.** 2007. Alanine Scanning of the Hepatitis C Virus Core Protein Reveals Numerous Residues Essential for Production of Infectious Virus. J Virol **81:**10220-10231.
4. **Jones CT, Murray CL, Eastman DK, Tassello J, Rice CM.** 2007. Hepatitis C Virus p7 and NS2 Proteins Are Essential for Production of Infectious Virus. J Virol **81:**8374-8383.
5. **Dentzer TG, Lorenz IC, Evans MJ, Rice CM.** 2009. Determinants of the Hepatitis C Virus Nonstructural Protein 2 Protease Domain Required for Production of Infectious Virus. Journal of Virology **83:**12702-12713.
6. **Pietschmann T KA, Koutsoudakis G, Shavinskaya A, Kallis S, Steinmann E, Abid K, Negro F, Dreux M, Cosset FL, Bartenschlager R.** 2006. Construction and characterization of infectious intragenotypic and intergenotypic hepatitis C virus chimeras. Proc Natl Acad Sci U S A **103:**7408-7413.
7. **Neveu G, Barouch-Bentov R, Ziv-Av A, Gerber D, Jacob Y, Einav S.** 2012. Identification and Targeting of an Interaction between a Tyrosine Motif within Hepatitis C Virus Core Protein and AP2M1 Essential for Viral Assembly. PLoS Pathog **8:**e1002845.
8. **Newton K, Matsumoto M, Ferrando R, Wickliffe K, Rape M, Kelley R, Dixit V.** 2012. Using Linkage-Specific Monoclonal Antibodies to Analyze Cellular Ubiquitylation, p 185-196. *In* Dohmen RJ, Scheffner M (ed), Ubiquitin Family Modifiers and the Proteasome, vol 832. Humana Press.
9. **Huang Y-P, Zhang S-L, Cheng J, Wang L, Guo J, Liu Y, Yang Y, Zhang L-Y, Bai G-Q, Gao X-S, Ji D, Lin S-M, Zhong Y-W, Shao Q.** 2005. Screening of genes of proteins interacting with p7 protein of hepatitis C virus from human liver cDNA library by yeast two-hybrid system. World Journal of Gastroenterology : WJG **11:**4709-4714.
10. **Erdtmann L, Franck N, Lerat H, Le Seyec J, Gilot D, Cannie I, Gripon P, Hibner U, Guguen-Guillouzo C.** 2003. The Hepatitis C Virus NS2 Protein Is an Inhibitor of CIDE-B-induced Apoptosis. Journal of Biological Chemistry **278:**18256-18264.
11. **de Chassey B, Navratil V, Tafforeau L, Hiet MS, Aublin-Gex A, Agaugué S, Meiffren G, Pradezynski F, Faria BF, Chantier T, Le Breton M, Pellet J, Davoust N, Mangeot PE, Chaboud A, Penin F, Jacob Y, Vidalain PO, Vidal M, André P, Rabourdin-Combe C, Lotteau V.** 2008. Hepatitis C virus infection protein network. Molecular Systems Biology **4**.
12. **Otsuka M, Kato N, Moriyama M, Taniguchi H, Wang Y, Dharel N, Kawabe T, Omata M.** 2005. Interaction between the HCV NS3 protein and the host TBK1 protein leads to inhibition of cellular antiviral responses. Hepatology **41:**1004-1012.

13. **Ding Q, Cao X, Lu J, Huang B, Liu Y-J, Kato N, Shu H-B, Zhong J.** Hepatitis C virus NS4B blocks the interaction of STING and TBK1 to evade host innate immunity. Journal of Hepatology **59:**52-58.
14. **Tong W-Y, Nagano-Fujii M, Hidajat R, Deng L, Takigawa Y, Hotta H.** 2002. Physical interaction between hepatitis C virus NS4B protein and CREB-RP/ATF6β. Biochemical and Biophysical Research Communications **299:**366-372.
15. **He Y SK, Tan SL.** 2006. HCV NS5A: A Multifunctional Regulator of Cellular Pathways and Virus Replication. . Horizon Bioscience, Norfolk (UK).
16. **Tu H, Gao L, Shi ST, Taylor DR, Yang T, Mircheff AK, Wen Y, Gorbalenya AE, Hwang SB, Lai MMC.** 1999. Hepatitis C Virus RNA Polymerase and NS5A Complex with a SNARE-like Protein. Virology **263:**30-41.
17. **Hirano M, Kaneko S, Yamashita T, Luo H, Qin W, Shirota Y, Nomura T, Kobayashi K, Murakami S.** 2003. Direct Interaction between Nucleolin and Hepatitis C Virus NS5B. Journal of Biological Chemistry **278:**5109-5115.
18. **Lu Q, Hope L, Brasch M, Reinhard C, Cohen S.** 2003. TSG101 interaction with HRS mediates endosomal trafficking and receptor down-regulation. Proc Natl Acad Sci U S A **100:**7626-7631.
