# Supplementary figures and images for: Hepatitis C Virus Proteins Interact with the Endosomal Sorting Complex Required for Transport (ESCRT) Machinery via Ubiquitination To Facilitate Viral Envelopment
Source: mBio. 2016 Nov 1;7(6):e01456-16. doi: 10.1128/mBio.01456-16 (PMC5090039; doi:10.1128/mBio.01456-16)

Fig. S1

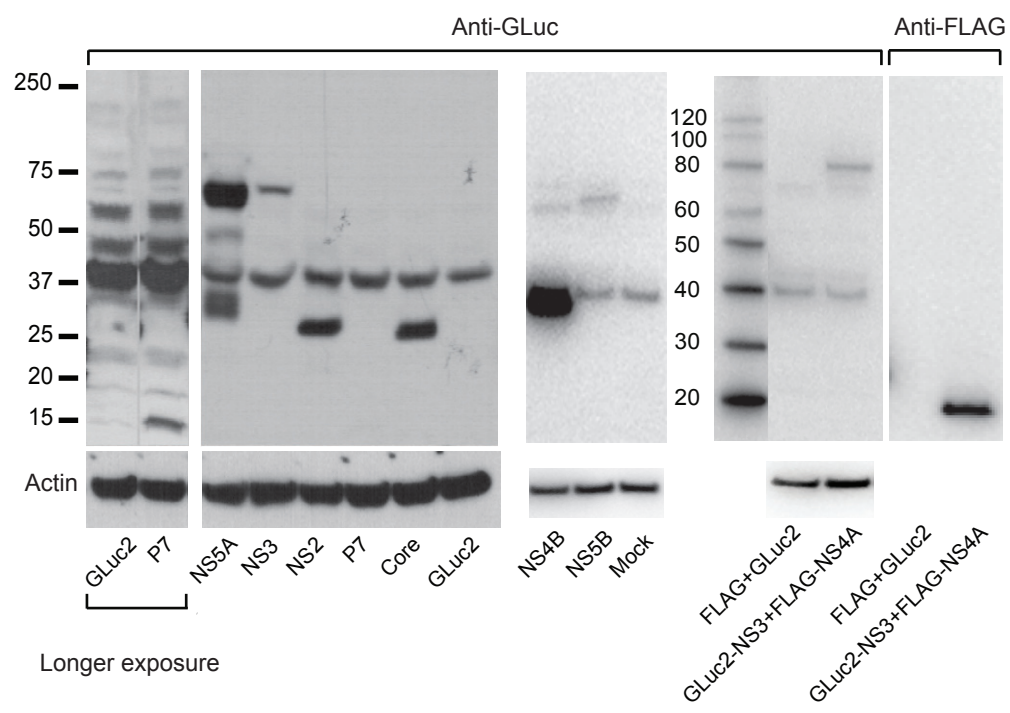

Supplement: Figure S1 — Expression of the viral proteins used in the PCA screen. 293T cells were transfected with plasmids encoding individual viral proteins fused to a GLuc2 tag or an empty GLuc2 plasmid. For NS3/NS4A coexpression (right panels), cells were cotransfected with GLuc2-NS3- and FLAG-NS4A-encoding plasmids or with the corresponding empty plasmids. Cell lysates collected at 24 h posttransfection were subjected to Western blot analysis using anti-GLuc, anti-FLAG, and anti-actin antibodies. Molecular mass marker mobility data are shown at the left in kilodaltons. Left panel: longer exposure of the control and P7 lanes. Download [file mbo005163053sf1.pdf]

Fig. S2

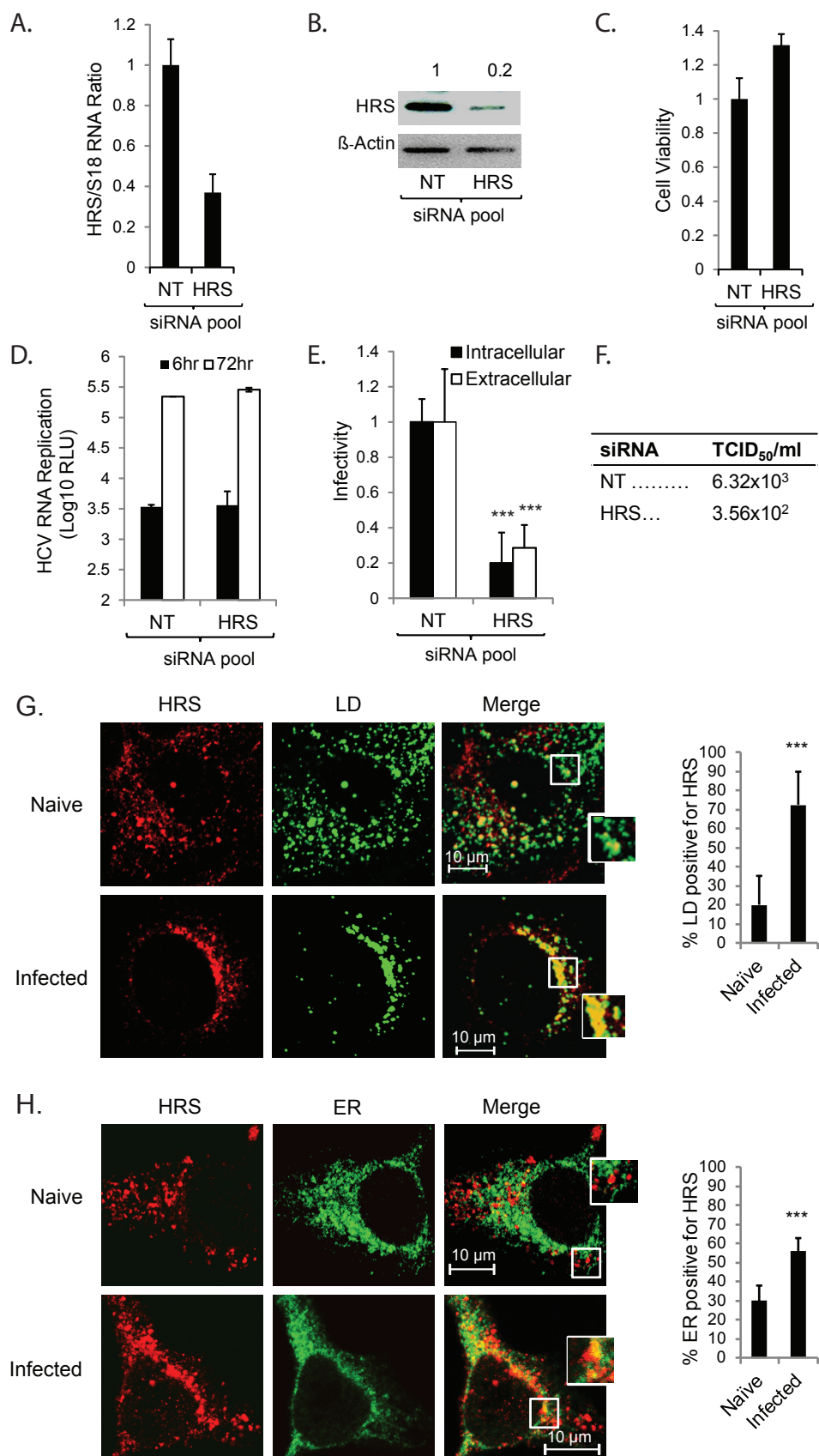

Supplement: Figure S2 — HRS is recruited to LDs and the ER to mediate HCV assembly. (A) HRS/S18 RNA ratio measured by qRT-PCR in Huh-7.5 cells transfected with a pool of four siRNAs (ON-TARGETplus SMARTpools; Dharmacon) targeting HRS or a pool of nontargeting (NT) sequences at 48 h posttransfection. (B) HRS protein levels determined by quantitative Western blotting in cells at 48 h posttransfection with the corresponding pooled siRNAs. Numbers represent HRS-to-actin protein ratios relative to the NT control. (C) Cellular viability determined by alamarBlue assays at 48 h posttransfection with siRNAs. Plotted data represent relative fluorescence values normalized to the NT control. (D) Cells were electroporated with J6/JFH(p7-Rluc2A) at 48 h posttransfection with the indicated pooled siRNAs. HCV RNA replication in these cells was determined by luciferase assays at 6 h (black) and 72 h (white) postelectroporation. (E) Intracellular (black) and extracellular (white) infectivity measured in naive Huh-7.5 cells infected with clarified cell lysates and supernatants derived from electroporated cells harboring the indicated siRNAs by luciferase assays, respectively. (F) Infectious virus production measured by limiting dilution assays. TCID50, 50% tissue culture infectious dose. (G and H) Representative images of HRS (red) and the LD marker Bodipy (G) or the ER marker calnexin (green) (H) in naive and HCV-transfected cells. Graphs represent percent colocalization (M2 values) of the indicated signals averaged from at least 20 cells for each category. Means ± SD (error bars) of results from at least two independent experiments are shown. RLU, relative light units. *, P < 0.05; **, P < 0.01; ***, P < 0.001 (Student’s t test). Download [file mbo005163053sf2.pdf]

Fig. S3

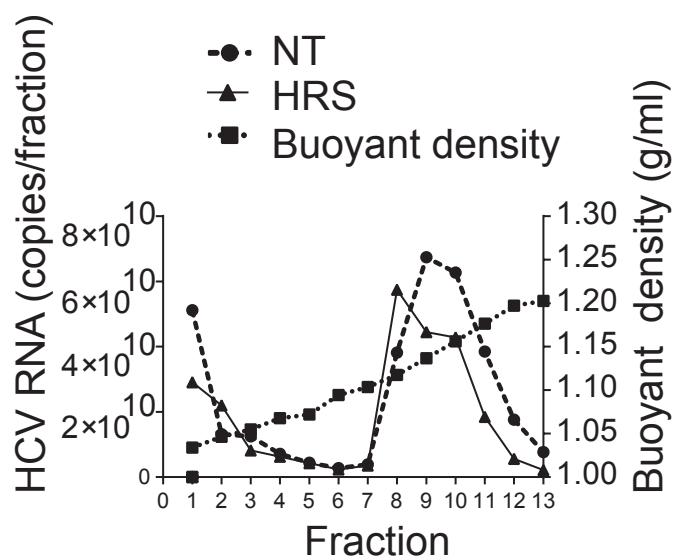

Supplement: Figure S3 — HRS depletion does not alter core cosedimentation with the HCV RNA. Clarified cell lysates derived from HCV-transfected HRS-depleted or control NT cell lines were layered on top of a continuous sucrose gradient (10% to 60%) and spun for 16 h at 36,000 rpm. A total of 13 fractions were collected and subjected to measurement of buoyant density by the use of a refractometer and of HCV RNA levels by qRT-PCR. Plotted data represent the buoyant density along the gradient (right axis) and the HCV RNA copy number per fraction (left axis). Download [file mbo005163053sf3.pdf]

Fig. S4

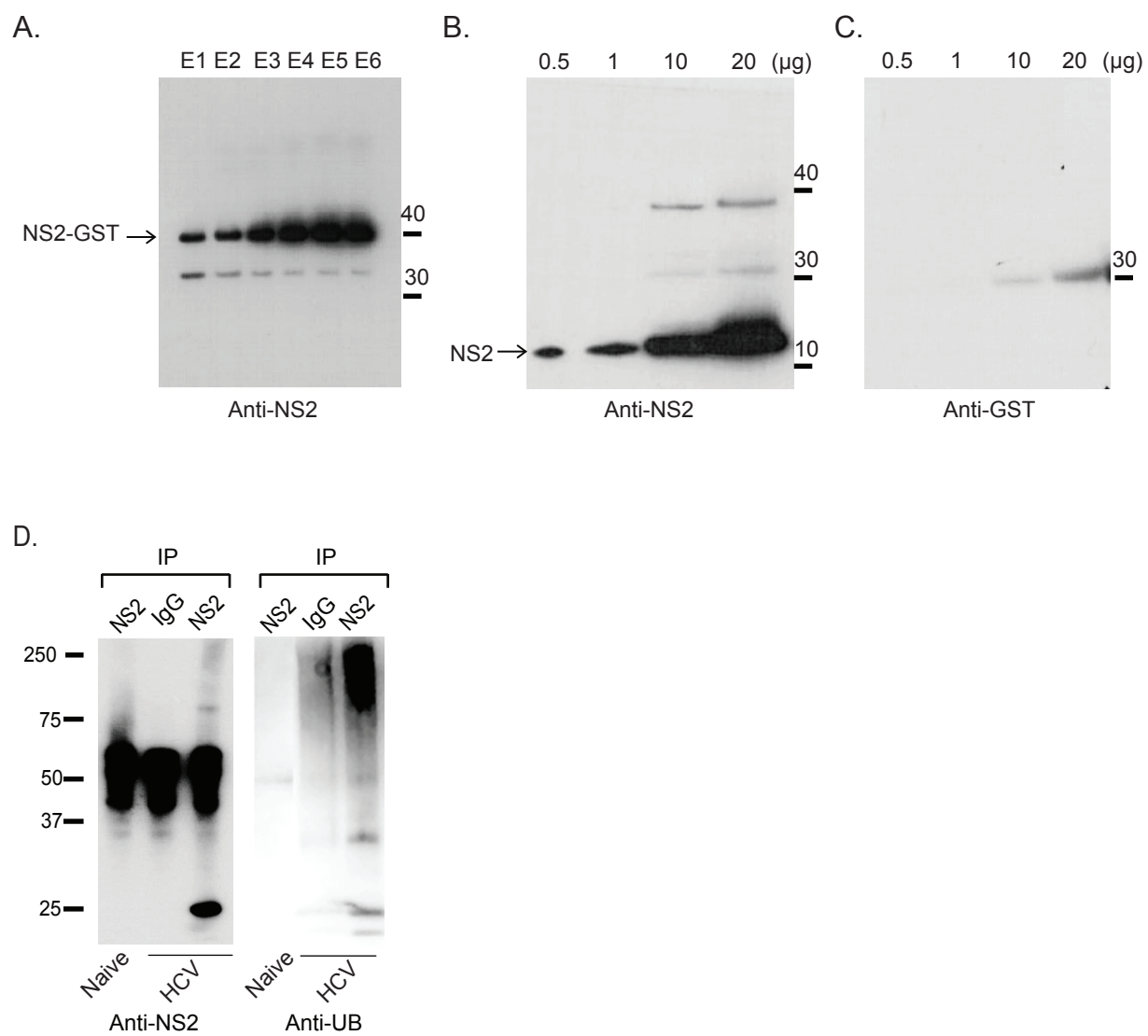

Supplement: Figure S4 — NS2 undergoes ubiquitination in vitro and in HCV-transfected cells. (A to C) Expression and purification of recombinant NS2. (A) Truncated NS2 protein (92 to 216 aa), fused to a C-terminal GST tag, was expressed in E. coli and purified on glutathione beads. Samples (5 µg) of protein from six elution fractions (E1 to E6) were separated by SDS-PAGE. NS2-GST was detected by immunoblotting with anti-NS2 antibodies. Following cleavage of the GST tag, protein samples (0.5 µg to 20 µg) were separated by SDS-PAGE. (B and C) Membranes were blotted with anti-NS2 (B) and anti-GST (C) antibodies. An ~14-kDa band corresponding to GST-cleaved truncated NS2 protein is shown in panel B. (D) Lysates of HCV RNA-transfected or naive Huh-7.5 cells were subjected to IP with anti-NS2 antibodies or IgG under denaturing conditions. Representative membranes blotted with anti-NS2 and anti-ubiquitin (UB) antibodies are shown. Download [file mbo005163053sf4.pdf]

Fig. S5

A.

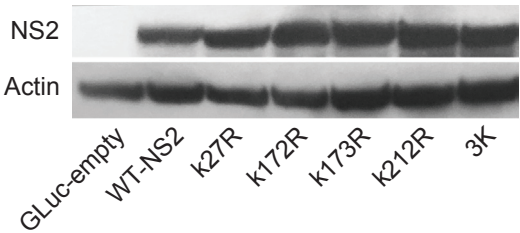

B.

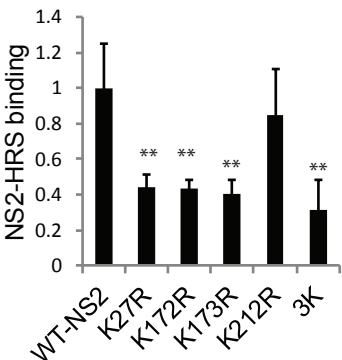

Supplement: Figure S5 — NS2 K-to-R mutations reduce HRS binding. (A) Levels of NS2 in lysates of cells transfected with the indicated plasmids. (B and C) Analysis of interactions of WT NS2 (B) or mutant NS2 with HRS (C) by PCAs. Plotted data represent NLRs relative to WT NS2-HRS binding. Mean values and standard deviations of results of 2 experiments (each performed in quadruplicate) are shown. **, P < 0.01 (Student’s t test). Download [file mbo005163053sf5.pdf]

Fig. S6

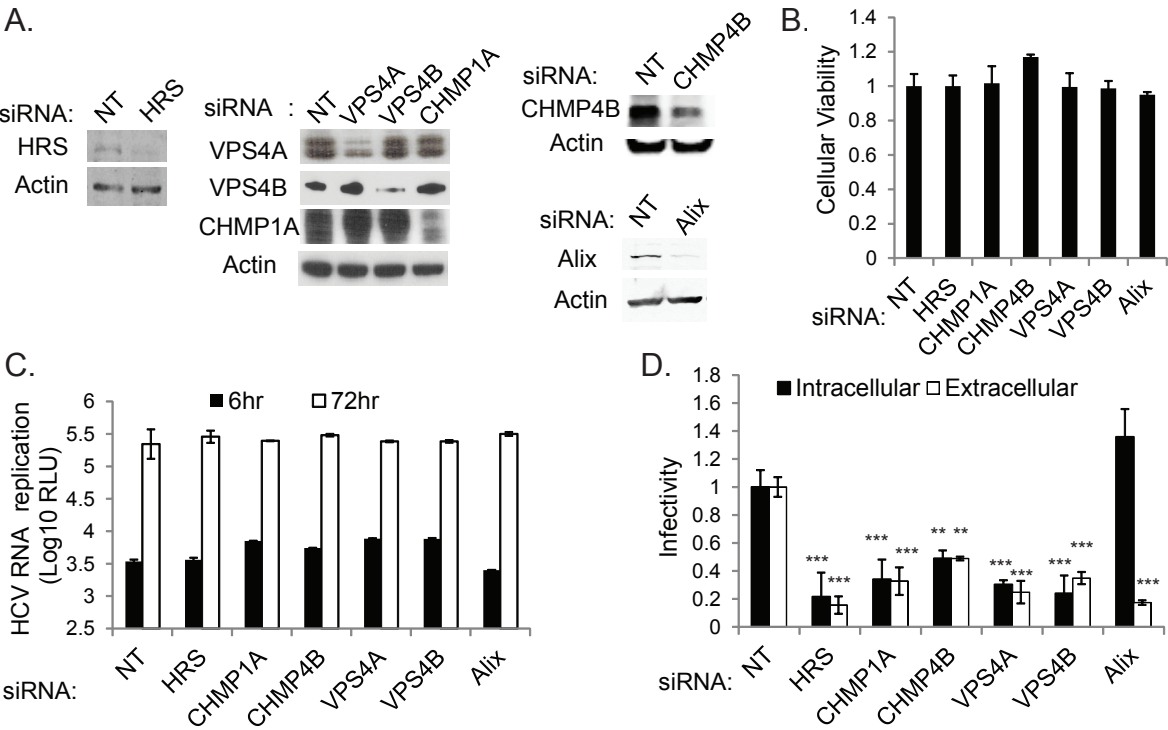

Supplement: Figure S6 — Additional ESCRT components mediate HCV assembly, whereas Alix mediates viral release. Huh-7.5 cells were transfected with the indicated siRNAs. (A) Representative membranes showing protein expression at 48 h posttransfection. (B) Cellular viability measured by alamarBlue-based assays and expressed as fluorescence values normalized to an NT control. (C) HCV RNA replication 6 and 72 h postelectroporation with in vitro transcribed HCV RNA measured by luciferase assays. (D) Intra- and extracellular infectivity by luciferase assays in naive cells inoculated with clarified cell lysates and supernatants derived from the HCV-electroporated cells, respectively. Plotted data represent infectivity normalized to NT controls. Mean values ± SD of results of 3 independent experiments are shown. **, P < 0.01; ***, P < 0.001 (Student’s t test). Download [file mbo005163053sf6.pdf]
