# Supplementary material for: Hepatitis C Virus Proteins Interact with the Endosomal Sorting Complex Required for Transport (ESCRT) Machinery via Ubiquitination To Facilitate Viral Envelopment
Source: mBio. 2016 Nov 1;7(6):e01456-16. doi: 10.1128/mBio.01456-16 (PMC5090039; doi:10.1128/mBio.01456-16)
Supplement: Table S1 — Analysis of ESCRT-HCV interactions by PCAs. The first and second columns list gene symbols and accession numbers. Numbers in parentheses represent several copies of the same open reading frame (ORF) in the human ORFeome library. The second to eighth columns list the average z scores measured by PCAs in three independent experiments each in triplicate. [file mbo005163053st1.pdf]

Table S1. ESCRT-HCV interactions by PCAs.

		Average Z-score							
Gene Symbol	Accession number	Core	P7	NS2	NS3	NS4A	NS4B	NS5A	NS5B
STAM2	BC028740	0.015676	-0.87888	1.242908	-0.21701	-0.5705	-0.22255	0.823636	0.257903
HRS (HGS)	BC003565	1.745720	2.26808	3.157132	0.028608	2.598538	2.088448	3.177182	1.434086
TSG101	BC002487	0.978705	-0.52204	0.356236	0.035954	0.322811	-0.36715	1.045919	0.566733
VPS28 (1)	BC006485	0.930497	-0.93367	0.448833	-0.15416	0.172568	-0.07173	1.071721	0.542738
VPS28 (2)	BC050713	1.141563	-0.72542	1.112355			1.126087	0.806110	0.623822
VPS37A (1)	BC022363	-0.24548	-1.23696	-0.141760	0.039051	-0.3859	0.564386	-0.167430	-0.02964
VPS37A (2)	BC067754	-0.34658	-0.72658	0.559987				0.023898	
VPS37B	BC005882	-0.48771	-0.91372	-0.894400	0.036569	-0.06401	-0.07868	0.172169	0.032803
EAP20	BC006282	1.13980	-0.70028	1.545432	-0.14616	0.647735	1.143455	1.267538	0.994738
EAP30 (1)	BC008976	1.005334	-0.86184	0.567178	-0.03613	-0.02766	-0.75545	0.666017	0.741755
EAP30 (2)	BC038830	1.118476	-0.84453	0.719862				0.646895	
CHMP1A	BC132711	2.664497	-0.02382	0.205736	-0.08462	0.878546	0.415478	1.152735	1.698734
CHMP1B	BC012733	0.274438	-1.09985	0.324901	-0.53597	-0.5369	0.019723	-0.181960	-0.3047
CHMP2A	BC002502	0.788947	-0.68396	-0.133890	-0.20861	0.455527	0.274951	0.843631	1.291945
CHMP2B	BC001553	0.255074	-0.70501	-0.737320	-0.49813	-0.25226	-0.61044	-0.301020	0.820115
CHMP3	BC004419	1.306319	-0.4086	0.139431	-0.19385	0.401511	0.440229	0.498064	1.103606
CHMP4A	BC010893	0.810694	-1.17724	-0.915160	-0.19525	-0.72572	-0.94102	0.167168	-0.04455
CHMP4B	BC033859	2.256789	-0.05645	0.437642	-0.14535	1.301781	0.551387	1.812682	1.246107
CHMP4C	BC014321	1.254212	-0.55187	0.119456	-0.48356	0.70787	0.395773	1.168513	0.920982
CHMP5 (1)	BC006974	1.757326	-0.71619	-0.295950	-0.06029	0.666511	-0.06803	1.050358	1.146025
CHMP5 (2)	BC007457	1.975911	-0.66587	-0.327370				0.980075	
CHMP5 (3)	BC016698	1.829131	-0.76255	-0.338680				0.995204	
CHMP6	BC010108	1.520425	-0.61842	0.112238	-0.40555	-0.73584	0.118734	1.781411	-0.41848
VPS4A	BC047932	1.628982	0.54400	0.628990	0.092851	0.583414	-0.23707	2.360545	1.366084
VPS4B	BC039574	1.193767	-0.11577	0.144372	-0.01901	-0.05435	-0.54783	0.922353	0.763869
VTA1	BC141827	0.603682	-0.97785	0.030349	0.107913	-0.32267	-1.1398	1.731984	0.942911
RPF1	BC016051	1.046517	-1.31691	-0.847280	-1.01345	-0.22958	-0.7572	-0.211310	0.173545
SLC9A6	BC049169	2.807277	1.000692	3.394888	-0.16306	1.447177	1.245898	0.937158	1.031942
ALIX (PDCD6IP)	BC020066	1.067352	-0.63400	0.684455	-0.02528	-0.09421	0.383872	1.835367	1.122457

The first and second columns list gene symbols and accession numbers. Numbers in parenthesis represent several copies of the same ORF in the human ORFeome library. The second to eighth columns list the average z-scores measured by PCAs in three independent experiments each in triplicates.
