# Supplementary material for: Hepatitis C Virus Proteins Interact with the Endosomal Sorting Complex Required for Transport (ESCRT) Machinery via Ubiquitination To Facilitate Viral Envelopment
Source: mBio. 2016 Nov 1;7(6):e01456-16. doi: 10.1128/mBio.01456-16 (PMC5090039; doi:10.1128/mBio.01456-16)
Supplement: Table S2 — Protein interactions of a random reference set (RRS) tested by PCAs to benchmark the screen. The first four columns list gene symbols and accession numbers of the interactors (A and B). The fifth column lists the average z scores measured by PCAs in three independent experiments each in triplicate. [file mbo005163053st2.pdf]

Table S2. Protein interactions of a Random Reference Set (RRS) tested by PCAs to benchmark the screen.

Gene Symbol (Interactor A)	Accession number (Interactor A)	Gene Symbol (Interactor B)	Accession number (Interactor B)	Average Z- Score
Clic4	BC012444	P21	BC000275	-0.28116
Clic4	BC012444	CD63	BC002349	0.48988
Clic4	BC012444	Sco1	BC015504	0.00597
Clic4	BC012444	TGIF	BC031268	-0.64097
Clic4	BC012444	ZFYV9	BC032680	0.26785
Clic4	BC012444	Cul2	BC009591	-0.29798
Clic4	BC012444	TCTP	BC003352	-0.22649
PLSL	BC007673	P21	BC000275	-1.82693
PLSL	BC007673	CD63	BC002349	-1.41779
PLSL	BC007673	Sco1	BC015504	-1.52319
PLSL	BC007673	TGIF	BC031268	-1.93038
PLSL	BC007673	ZFYV9	BC032680	-1.00535
PLSL	BC007673	Cul2	BC009591	-1.39101
PLSL	BC007673	TCTP	BC003352	-1.83576
GPR87	BC009540	P21	BC000275	-0.89406
GPR87	BC009540	CD63	BC002349	1.82948
GPR87	BC009540	Sco1	BC015504	-0.79888
GPR87	BC009540	TGIF	BC031268	-0.97016
GPR87	BC009540	ZFYV9	BC032680	-0.72268
GPR87	BC009540	Cul2	BC009591	-0.87390
GPR87	BC009540	TCTP	BC003352	-0.79490
SNP1	BC027040	P21	BC000275	0.79422
SNP1	BC027040	CD63	BC002349	0.86158
SNP1	BC027040	Sco1	BC015504	0.78750
SNP1	BC027040	TGIF	BC031268	1.56015
SNP1	BC027040	ZFYV9	BC032680	-0.32781
SNP1	BC027040	Cul2	BC009591	0.73479
SNP1	BC027040	TCTP	BC003352	-0.46141
SYND4	BC030805	P21	BC000275	-0.62872
SYND4	BC030805	Sco1	BC015504	0.41904
SYND4	BC030805	TGIF	BC031268	-0.93836
SYND4	BC030805	ZFYV9	BC032680	-0.50770
SYND4	BC030805	Cul2	BC009591	-0.26543
SYND4	BC030805	TCTP	BC003352	-0.23992
DDIT3	BC003637	P21	BC000275	0.16148
DDIT3	BC003637	CD63	BC002349	1.87110
DDIT3	BC003637	Sco1	BC015504	1.45736
DDIT3	BC003637	TGIF	BC031268	0.71079
DDIT3	BC003637	ZFYV9	BC032680	0.51147
DDIT3	BC003637	Cul2	BC009591	0.96960
DDIT3	BC003637	TCTP	BC003352	0.40707

Gene Symbol (Interactor A)	Accession number (Interactor A)	Gene Symbol (Interactor B)	Accession number (Interactor B)	Average Z- Score
SNX15	BC009896	P21	BC000275	1.20272
SNX15	BC009896	CD63	BC002349	1.44127
SNX15	BC009896	Sco1	BC015504	1.46132
SNX15	BC009896	TGIF	BC031268	1.15180
SNX15	BC009896	Cul2	BC009591	1.40228
SNX15	BC009896	TCTP	BC003352	1.00368
CKLF1	BC009942	P21	BC000275	-0.31429
CKLF1	BC009942	Sco1	BC015504	0.12415
CKLF1	BC009942	TGIF	BC031268	-0.46713
CKLF1	BC009942	ZFYV9	BC032680	-0.12612
CKLF1	BC009942	Cul2	BC009591	-0.01303
CKLF1	BC009942	TCTP	BC003352	0.09578

The first four columns list gene symbols and accession numbers of the interactors (A and B). The fifth column lists the average z-scores measured by PCAs in three independent experiments each in triplicates.
