# Supplementary material for: Hepatitis C Virus Proteins Interact with the Endosomal Sorting Complex Required for Transport (ESCRT) Machinery via Ubiquitination To Facilitate Viral Envelopment
Source: mBio. 2016 Nov 1;7(6):e01456-16. doi: 10.1128/mBio.01456-16 (PMC5090039; doi:10.1128/mBio.01456-16)
Supplement: Table S3 — Known positive protein interactions tested by PCAs. The first four columns list gene symbols and accession numbers of the interactors (A and B). The fifth column lists the average z scores measured by PCAs in three independent experiments each in triplicate. [file mbo005163053st3.pdf]

Table S3. Known positive protein interactions tested by PCAs.

Gene Symbol (Interactor A)	Accession number (Interactor A)	Gene Symbol (Interactor B)	Accession number (Interactor B)	Average Z-Score	Reference
Core	--	AP2M1	BC013796	2.17	(7)
P7	--	MS4A6A	BC022854	2.31	(10)
NS2	--	CIDEB	BC035970	1.45	(11)
NS3/NS4A	--	GFAP	BC013596	1.86	(12)
NS3/NS4A	--	TBK1	BC034950	2.54	(13)
NS4A	--	CREB3	BC010158	1.44	(12)
NS4B	--	STING	BC047779	2.2	(14)
NS4B	--	ATF6B	BC008394	1.44	(15)
NS5A	--	VAPA	BC002992	3.24	Reviewed in (16)
NS5A	--	RAF1	BC018119	2.35	Reviewed in (He Y, 2006)
NS5A	--	GRB2	BC000631	3.06	Reviewed in (He Y, 2006)
NS5A	--	PITX	BC003685	2.04	Reviewed in (He Y, 2006)
NS5A	--	P53	BC003596	2.005	Reviewed in (He Y, 2006)
NS5B	--	hVAP33	BC002992	3.19	(17)
NS5B	--	NCL	BC002343	1.68	(18)
HRS	BC003565	TSG101	BC002487	2.30	(19)
SMAD4	BC002379	SKIL	BC059386	6.34	(2)

The first four columns list gene symbols and accession numbers of the interactors (A and B). The fifth column lists the average z-scores measured by PCAs in three independent experiments each in triplicates.
